# Health Outcomes of Construction Workers Building Infrastructure for Mega-Sporting Events: A Systematic Review of the Literature

**DOI:** 10.3390/ijerph22010004

**Published:** 2024-12-24

**Authors:** Davide J. Testa, João P. Vale, Leonidas G. Ioannou, Petros C. Dinas, Tiago S. Mayor, Kristine H. Onarheim, Zahra R. Babar, Sally Hargreaves, Andreas D. Flouris

**Affiliations:** 1 Department of Physical Education and Sport Science, University of Thessaly, Karies, 42100 Trikala, Greeceleonidas@heat-health.org (L.G.I.); pentinas@pe.uth.gr (P.C.D.); 2 CEFT, Transport Phenomena Research Centre, Faculty of Engineering, University of Porto, Rua Dr. Roberto Frias, 4200-465 Porto, Portugal; 3 ALiCE, Associate Laboratory in Chemical Engineering, Faculty of Engineering, University of Porto, Rua Dr. Roberto Frias, 4200-465 Porto, Portugal; 4 Department of Global Public Health and Primary Care, University of Bergen, Postboks 7804, NO-5020 BERGEN, 5007 Bergen, Norway; kristine.onarheim@uib.no; 5 Center for International and Regional Studies, Georgetown University, Doha P.O. Box 23689, Qatar; zb36@georgetown.edu; 6 Migrant Health Research Group and the Consortium for Migrant Worker Health, City St George’s, Institute for Infection and Immunity, University of London, London SW17 0RE, UK; shargrea@sgul.ac.uk

**Keywords:** mega-sporting events, FIFA World Cup, Olympic Games, Commonwealth Games, Asian Games, migrant workers, construction labor, labor violations, health outcomes

## Abstract

Background: Migrant construction workers involved in building infrastructure for mega-sporting events face elevated risks of illness and death. However, specific health outcomes for these workers have not been systematically reviewed, limiting opportunities to identify and address their challenges. Methods: This study systematically reviewed health outcomes among migrant construction workers involved in mega-sporting events. Results: 89 eligible studies involving 23,307 workers were identified. Of these, only 11 directly addressed specific health outcomes, including heat stress, occupational fatalities, and sexually transmitted infections. Notably, increased heat exposure during peak construction phases and the proximity of deadlines for mega-sporting events were correlated with elevated rates of occupational fatalities. Other key adverse factors impacting migrant construction workers’ health included an observed correlation between the timing of mega-sporting events and increased occupational fatalities, the involvement of labor recruiters, and shifting health and safety responsibilities among stakeholders (e.g., host states, event organizers, contractors, and recruitment agencies). Positive outcomes were observed when workers voluntarily engaged in non-mandatory safety activities, such as safety training programs and awareness meetings. Conclusions: There is a critical need for longitudinal and comparative studies to comprehensively examine the health of migrant workers throughout all stages of their journey, from pre-migration to return. This review underscores the urgency of prioritizing evidence-based policies that address unique health risks in this population, including mitigation of heat stress and enforcement of occupational safety standards, particularly amid construction spikes preceding mega-sporting events. Recommendations: Future research should prioritize understanding the unique health challenges faced by migrant workers to inform policy making, develop effective interventions, and implement best practices to improve their health and well-being.

## 1. Introduction

The migrant labor force—who are especially vulnerable to exploitation and discrimination and are less protected by domestic labor laws—is currently at approximately 169 million and growing [1]. Indeed, hundreds of thousands of construction workers are involved in building the infrastructure required to host mega-sporting events such as the Fédération Internationale de Football Association (FIFA) World Cup [2]. Given constraints in labor supply in host countries, the majority of such jobs are usually filled by low-skilled, low-waged temporary migrant laborers [3]. However, investigative journalists [2,4] have recently exposed cases of forced labor and higher-than-sector-average casualty rates among workers involved in the construction for mega-sporting events. For example, reports from Qatar’s 2022 FIFA World Cup construction projects revealed thousands of deaths among migrant workers, with many attributed to preventable causes such as heat stress and unsafe working conditions [2,5].

Mega-sporting event construction projects differ from typical construction projects in several ways that contribute to elevated health risks for workers. First, these projects often operate under extreme time constraints and rigid deadlines, leading to rushed work processes and the bypassing of critical safety protocols [3,6]. Second, the intermittent and short-term nature of these events means that many workers are employed on short-term contracts or temporary visas, which limit their access to healthcare, legal protections, and compensation for injuries [7]. Third, the intense global scrutiny surrounding these events incentivizes host countries and contractors to prioritize project completion over worker well-being, often resulting in exploitative labor practices such as excessive working hours, inadequate rest periods, and poor living conditions [2,4]. Collectively, these factors create a work environment with unique and compounded risks, making mega-sporting event construction projects particularly hazardous for workers.

The health outcomes of construction workers have been the object of study of academics and not-for-profit organizations [5,8]. There is a consensus that these workers face an elevated risk of occupational injuries and fatalities when compared to their counterparts in other sectors [5,6]. Recently, a systematic review and meta-analysis focusing on the occupational health of international migrant workers [5] highlighted that migrant construction workers often endure body aches, joint paints, and injuries, while receiving low wages and working long hours. Moreover, these factors, namely engaging in construction work, experiencing physical illnesses, dealing with low wages, and enduring long working hours, were all found to be associated with a higher prevalence of depression in this population. However, while the aforementioned study shed light on the challenges faced by migrant construction workers, it did not specifically analyze the health outcomes of those involved in building infrastructure for mega-sporting events. Unlike other migrant construction workers, those engaged in mega-sporting event projects face unique circumstances due to the intermittent and short-term nature of these events, as well as the short-term visas and specific occupational statuses they hold [3,6]. As a consequence, there is a pressing need to systematically scrutinize this subgroup of construction workers, as their distinct circumstances may hinder the understanding of their health challenges and implementation of effective initiatives aimed at safeguarding their health [6,7].

Currently, no systematic reviews have specifically focused on the health outcomes of individuals involved in the construction of mega-sporting events. This study aims to bridge this knowledge gap by systematically analyzing the existing literature on the health implications faced by individuals working on mega-sporting event infrastructure projects. To achieve this goal, the study encompassed two searches. The first review focused on the health outcomes of people involved in construction for mega-sporting events. Because these people were found by previous literature to consist predominantly of migrant workers [3], the first review was complemented by a second review on violations, recruitment, and/or workplace practices associated with migrant construction workers. In doing so, we sought to comprehensively inform on the quality of the available evidence, risk factors, and preventive strategies that can be implemented to protect this vulnerable population in future guidelines and policies.

## 2. Materials and Methods

### 2.1. Protocol Registration, Reporting, Ethical Approval, and Patient and Public Involvement Statement

We preregistered the protocol of this systematic review of the literature in the Open Science Framework registries (OSF) (preregistration link https://doi.org/10.17605/OSF.IO/W4RPU) and reported this study in accordance with the Preferred Reporting Items for Systematic Reviews and Meta-analyses (PRISMA) checklist available in Table S1 in the Supplementary File [9]. Ethical approval was not required for this review as all the collected information was available to the public. No patients or members of the public were involved in this research.

### 2.2. Search Strategy

We searched the Embase, PubMed, Ovid MEDLINE, and Scopus databases from the date of their inception to 13 April 2022, with no limitation for study design, peer-review status, or language, in order to find articles investigating health outcomes of construction workers involved in building infrastructure for mega-sporting events (search 1). Furthermore, we searched the Embase, PubMed, Ovid MEDLINE, Scopus, and Google Scholar databases from the date of their inception to 13 April 2022, with no limitation for study design, peer-review status, or language, with the aim of complimenting the previous search by targeting articles investigating violations, recruitment, and/or workplace practices relevant to migrant construction workers (search 2). Following the database searches, we manually searched the reference lists of the database-retrieved articles. The inclusion and exclusion criteria for eligibility are listed in Table 1. The search algorithms used for the database searches are provided in the Supplementary File.

### 2.3. Eligibility Criteria

Articles were found to be eligible for search 1 if they addressed the health outcomes of persons involved in the construction for mega-sporting events. Articles were eligible for search 2 if they addressed violations, recruitment, and/or workplace practices relevant to migrant construction workers (see Supplementary File). We initially removed duplicates and screened all the unique articles for title and abstract against the eligibility criteria (see Supplementary File) and later screened the full text of the articles that were found to be potentially eligible during the title-and-abstract screening against the same eligibility criteria (literature reviews were deemed an eligible article type, see Supplementary File). When the full text of a study was not available online, we requested the full text from its authors. Title, authors’ names and surnames, digital object identifier (DOI), and abstract of all retrieved articles were imported, when available, into Excel spreadsheets (Microsoft Office, Microsoft, Washington, the US) where the title-and-abstract and full-text screenings, quality appraisal, and data extraction were performed. The eligibility screening was conducted independently by five investigators (DJT, JPV, PCD, ADF, LGI) and conflicts were resolved through consensus.

### 2.4. Quality Appraisal

We used the Research Triangle Institute (RTI) item bank checklist [10] to appraise the quality of observational studies while we used the Scale for the Assessment of Narrative Review Articles (SANRA) [11] for the appraisal of narrative review articles, in line with previous literature [12,13]. Quality appraisal was undertaken independently by three investigators (DJT, JPV, LGI) and conflicts were resolved through consensus.

### 2.5. Data Extraction

For all eligible studies, we extracted the first author’s surname and country affiliation, publication year, study design, main outcome measures, countries of focus, funding received, sports event of focus, and participants’ number, age, countries of origin, and occupation. In search 1, we additionally extracted the health hazards addressed and health outcomes observed. In search 2, we also extracted the addressed violations, recruitment and workplace practices, and their observed effects on health outcomes, if any were observed. The data extraction was conducted by three investigators (DJT, JPV, ADF) and conflicts were resolved through consensus.

### 2.6. Evidence Synthesis

We refrained from conducting meta-analyses due to the significant variability in outcome measures and intervention types among eligible studies [14]. Thus, a narrative data synthesis was conducted, following the Synthesis Without Meta-analysis (SWiM) reporting guidelines that are intended to complement and be used as an extension to PRISMA [15] (see Table S2 in the Supplementary File). In line with the aims of this review, we synthesized the evidence collected by first grouping the eligible articles on whether they reported health outcomes of construction workers involved in building infrastructure for mega-sporting events (search 1) or recruitment practices, workplace practices, or violations relevant to migrant construction workers (search 2). We subsequently conducted a vote-counting assessment [16] to identify the direction of the observed effects (either negative, null, or positive) of working in the construction for mega-sporting events, recruitment practices, workplace practices, and violations on the health outcomes of construction workers. The vote-counting was conducted by two independent investigators (DJT, JPV) and conflicts were resolved through consensus. The results were presented, listing, for each eligible article, the first author’s surname and publication year, study design, sample characteristics and size, risk of bias, intervention, and outcome measures. For the studies eligible for search 2, we categorized the extracted intervention measures between violations and non-violations based on how they were defined in the source study. The studies were ordered alphabetically by the first authors’ surname in all tables. The heterogeneity in observed effects was examined using descriptive statistics and harvest plots [16].

The narrative synthesis focuses on the studies specifically measuring the effect of health hazards on health outcomes of migrant construction workers. A full reference list of all the 89 articles eligible to search 1 and 2 is included in the Supplementary File.

## 3. Results

### 3.1. Searching and Selection Screening Outcomes

**Figure 1:**
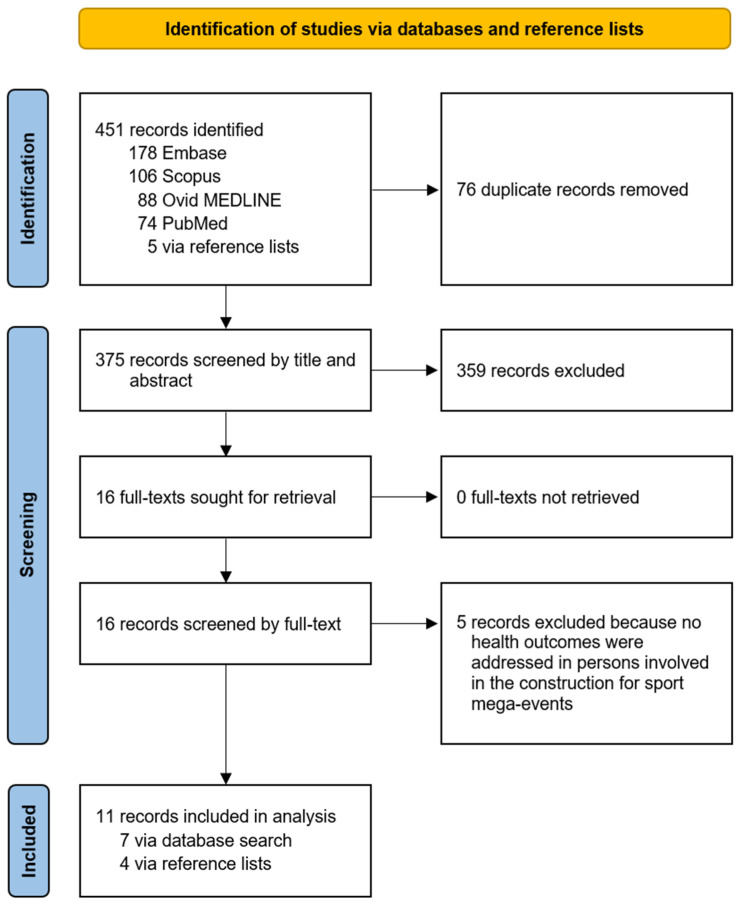
Study selection for search 1.

**Figure 2:**
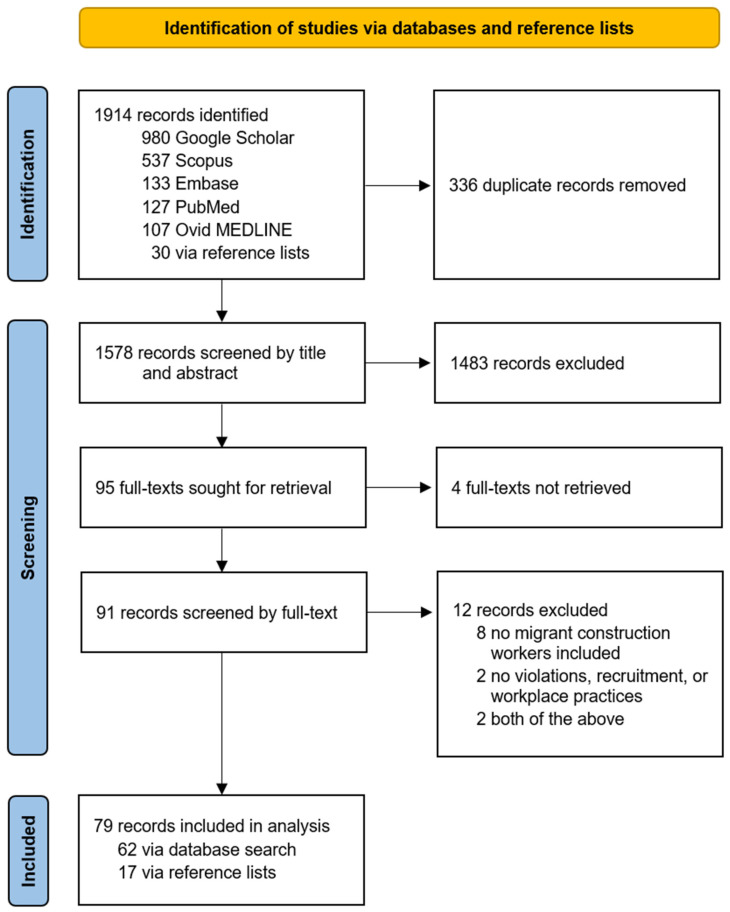
Study selection for search 2.

A total of 451 articles were identified through search 1 and 1914 through search 2. For search 1, 76 duplicates were removed, and 375 unique articles underwent title-and-abstract screening. Of these, 16 articles were found to be potentially eligible and were full-text screened (Figure 1). For search 2, 336 duplicates were removed, and 1578 unique articles underwent title-and-abstract screening. Of these, 91 articles were found to be potentially eligible and were full-text screened (Figure 2). As a result, 11 articles were found eligible through search 1 and 79 through search 2 (Figure 1 and Figure 2). One article [17] was found to be eligible through both searches. In total, 89 articles were considered eligible.

### 3.2. Characteristics of All the Eligible Studies

The 89 eligible studies were published between 1992 and 2021 and included 41,745 participants, out of which at least 23,307 were construction workers. The remaining participants consisted of academics, employers and delegated managers, government officials, journalists, labor recruiters, lawyers, staff of non-governmental and inter-governmental organizations and trade union representatives, among others. Of the eligible studies, 21 (23.3%) focused on the US, 13 (14.4%) on Qatar, 10 (11.2%) on China, 8 (8.9%) on India, and 6 (6.7%) on the UK while 29 studies (32.2%) focused on other countries and 3 (3.3%) focused on no country in particular (Table 2). The vast majority of studies (68 equaling to 75.6% of all studies) had a cross-sectional design and only five (5.6%) had a longitudinal design. In addition, 13 studies (14.4%) were literature reviews, 2 (2.2%) had a retrospective design, and 2 (2.2%) had a mixed design. 37 studies (41.1%) received governmental funding to conduct their research, while 35 (38.9%) did not report funding, 7 (7.7%) received public donations, 5 (5.6%) received funding from foundations or charities, 5 (5.6%) from many different sources and 1 (1.1%) from an inter-governmental organization. Overall, 21 studies (23.3%) addressed health outcomes in construction workers, including all the 11 studies eligible for search 1 as well as 11 of the studies eligible for search 2, considering that one such study was eligible to both searches [17]. The full list of studies included in the systematic review for search 1 and 2 is provided in the Supplementary File.

### 3.3. Risk of Bias Assessment Outcomes

Most studies (59 equaling to 65.5% of all studies) were found to have an unclear risk of bias, commonly due to a lack of relevant information within the full-text articles, while 17 (18.9%) had a low risk of bias, 7 (7.8%) a moderate risk, and 7 (7.8%) a high risk of bias. Of the articles, 66 (73.3%) were peer-reviewed while the remaining 24 (26.7%) were not peer-reviewed.

Transitioning to the next section, the focus shifts to the specific health outcomes associated with working at mega-sporting events.

### 3.4. Effect of Working at Mega-Sporting Events on Health Outcomes (Search 1)

All 11 studies eligible for search 1 (Table 3) addressed health outcomes in persons involved in the construction for mega-sporting events: three studies (27.3%) [7,18,19] had a low risk of bias, three (27.3%) [6,17,20] moderate, three (27.3%) [21,22,23] unclear, and two (18.1%) [24,25] high. However, only five of these studies (45.5%) [6,17,19,21,24] actually measured the effect of health hazards on health outcomes, while the remaining six (54.5%) assessed the prevalence of infectious diseases among construction workers [18,22], identified occupational health hazards [23], reported health management practices [25], examined the literature on the well-being and engagement measures for construction workers [20], or produced health policy recommendations for improving the health of migrant construction workers [7].

**Figure 3:**
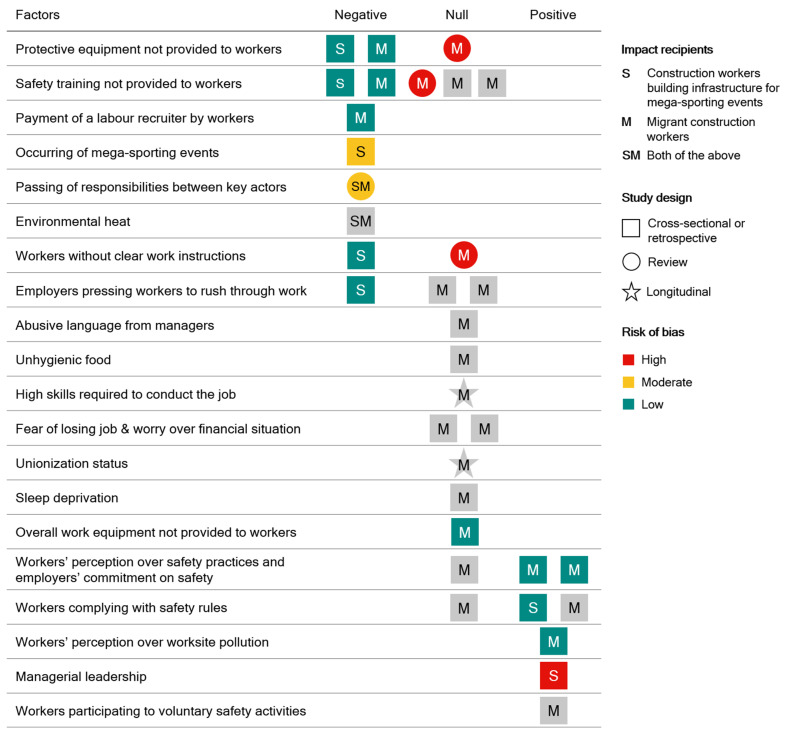
Impact of factors found by the studies eligible for search 1 and 2 on health outcomes of construction workers and/or migrant construction workers involved in building infrastructure for mega-sporting events. Key factors include: protective equipment not provided to workers (Katsakiori 2008 [19], Zerguine 2018 [26], Anand 1998 [27]), safety training not provided to workers (Katsakiori 2008 [19], Zerguine 2018 [26], Amnesty 2009 [8], Anand 1998 [27], Roelofs 2011 [28]), payment of a labour recruiter by workers (Hassan 2014 [29]), occurring of mega-sporting events (Flouris 2021 [6]), passing of responsibilities between key actors (Millward 2017 [17]), environmental heat (Flouris 2019 [21]), workers without clear work instructions (Katsakiori 2008 [19], Anand 1998 [27]), employers pressing workers to rush through work (Katsakiori 2008 [19], Dutta 2017 [30], Roelofs 2011 [28], abusive language from managers (Dutta 2017 [30]), unhygienic food (Dutta 2017 [30]), high skills required to conduct the job (Anderson 2000 [31]), fear of losing job & worry over financial situation (Dutta 2017 [30], Roelofs 2011 [28]), unionization status (Anderson 2000 [31]), sleep deprivation (Dutta 2017 [30]), overall work equipment not provided to workers (Zerguine 2018 [26]), workers’ perception over safety practices and employers’ commitment on safety (Chan 2017 [32], Zerguine 2018 [26], Zerguine 2018 [26]), workers complying with safety rules (Chan 2017 [32], Katsakiori 2008 [19], Lyu 2018 [33]), workers’ perception over worksite pollution (Jiang 2020 [34], managerial leadership [Shiplee 2011 [24]), and workers participating to voluntary safety activities (Lyu 2018 [33]).

Four out of the five studies measuring an effect (80.0%) focused on factors that were found to worsen the health outcomes of persons involved in construction for mega-sporting events (Figure 3). In particular, Flouris and colleagues (2019) [21] focused on the threat posed by environmental heat on the health of 125 migrant workers building infrastructure for the 2022 FIFA World Cup and laboring in agriculture in Qatar in different working scenarios. They found that participants operating in the business-as-usual scenario spent, on average, 30.0% of their working time at borderline-hyperthermic levels (i.e., core temperature between 37.5 and 37.9 °C), while 5.0% of their working time was spent at hyperthermic levels (i.e., between 38.0 and 38.4 °C) placing them at risk to succumb to heat-induced illnesses such as heat exhaustion and heatstroke. At the same time, the authors found that the average levels of occupational heat strain experienced by the participants were similar to those observed by other studies conducted in countries outside the Gulf region, due to the fact that the participants spent on average nearly half of their working time in unplanned breaks as a mechanism to cope against the high heat and humidity. Also, Millward (2017) [17] focused on the health of migrant workers employed for the building of infrastructure for the 2022 FIFA World Cup in Qatar. They focused on the passing of responsibilities between the Government of Qatar, FIFA, World Cup sponsors, building contractors and sub-contractors, and recruitment agencies on the occupational injuries and deaths suffered by migrant workers. They found that all the mentioned actors framed the injuries and deaths as regrettable situations but unconnected to their own work and were therefore unwilling to redress the causes that led to health damages and could cause more in the future.

Katsakiori and colleagues (2008) [19] aimed at identifying the factors that caused occupational fatalities among construction workers in Athens in Greece during the five years preceding the 2004 Olympic and Paralympic Games in Greece. They found that the causing factors were primarily under the responsibility of employers and delegated managers, such as the missed provision of appropriate protective equipment and clear information on job assignments to laborers, as well as an excessive time pressure in relation to deadlines, among others. On a similar note, Flouris and colleagues (2021) [6] assessed the incidence of occupational fatalities among construction workers in the seven years before and one year after the Olympic and Paralympic Games in Barcelona (Spain) in 1992, in Atlanta (the US) in 1996, in Sidney (Australia) in 2000, in Athens (Greece) in 2004, in London (the UK) in 2012, and in Rio the Janeiro (Brazil) in 2016. Importantly, this analysis showed an increase in the incidence of occupational fatalities among construction workers in the five years before each of the Games opened.

Only one out of the five studies measuring an effect (20.0%) found factors exerting a positive effect on the health outcomes of construction workers. This was the case of Shiplee and colleagues (2011) [24] who reported on the health and safety measures planned to be implemented for the protection of construction workers building infrastructure for the 2012 Olympic and Paralympics Games of London, UK. The authors concluded that the construction sites for the games saw lower accident rates compared to the average of the British construction sites.

Overall, the above-mentioned studies assessed the effect of nine different factors (Figure 3) on the health outcomes of construction workers involved in building infrastructure for mega-sporting events (search 1), with eight factors (88.9%) found to worsen health outcomes and one (11.1%) to improve them.

Transitioning to the second search, we also identified 21 additional factors related to the effect of recruitment and workplace practices and violations on the health outcomes of migrant construction workers (search 2). These factors are illustrated in Figure 3 and are detailed in the following sections. Descriptive information about studies investigating violations, recruitment, and/or workplace practices relevant to migrant construction workers is provided in the Supplementary File (Table S3).

### 3.5. Effect of Recruitment Practices on Health Outcomes (Search 2)

Only one study (1.3%) [29] in search 2 assessed the effect of recruitment practices on the health outcomes of migrant construction workers. The study in question had a low risk of bias. It assessed if Indian migrant workers indebted with recruitment agents in their home country in order to find employment in the Middle East were more likely to have suffered a workplace accident while abroad compared to the Indian migrant workers that did not pay recruitment agents. The study found that skilled workers that had paid a labor recruiter were statistically significantly more likely to have had suffered a worksite accident while no statistically significant differences were found for unskilled workers and supervisors.

### 3.6. Effect of Workplace Practices on Health Outcomes (Search 2)

Nine studies (11.4%) [8,26,27,28,30,31,32,33,34] in search 2 assessed the effect of workplace practices on the health outcomes of migrant construction workers. Of these, six studies (66.7%) [8,28,30,31,32,33] had an unclear risk of bias, two (22.2%) [26,34] low, and one (11.1%) [27] high. Overall, the nine studies addressed 12 different workplace practices, out of which nine (75.0%) were found to not affect health outcomes, two (16.7%) to improve them, and one (8.3%) to exert mixed effects. Specifically, null effects on the health outcomes of migrant construction workers were found for the following practices: employers failing to provide clear work instructions to workers [27] or safety training in a language in which the workers were fluent [8], workers rushing through their work as a reaction to pressures put on them by employers and delegated managers aiming to increase productivity [28,30], delegated managers using an abusive language toward workers [30], workers suffering from sleep deprivation [30] or receiving unhygienic food [30], workers’ unionization [31], fears of losing the job and worries about their own financial situation [28,30], and the level of skill required to conduct the work [31]. Importantly, we oftentimes rated as null the effect of the above factors on health outcomes because the design of the relevant studies did not have enough power to prove an actual effect (e.g., studies did not include statistical analyses or control groups necessary to justify their conclusions). The factors found to improve health outcomes in migrant construction workers were workers’ perceptions over worksite air, noise, and industrial waste pollution [34], as well as participation in voluntary activities at the worksite, aimed at improving overall worksite safety, such as attending non-mandatory meetings on safety and helping fellow workers [33]. Migrant workers’ perception of safety practices and employers’ commitment to safety was found to exert mixed effects on workers’ health outcomes. Chan and colleagues (2017) found a null effect and Zerguine and colleagues (2018) found positive effects of workers perceiving that their company of employment had an ‘interest’ in workers’ health and safety and was committed to safeguarding workers’ health.

### 3.7. Effect of Violations on Health Outcomes (Search 2) 

Six studies (7.6%) [17,26,27,28,32,33] in search 2 assessed the effect of violations on the health outcomes of migrant construction workers. Of these, three studies (50.0%) [28,32,33] had an unclear risk of bias, one (16.7%) [26] low, one (16.7%) [17] moderate, and one (16.7%) [27] high. Overall, the six studies addressed five different violations. Three (60.0%) were found to have mixed effects on the health outcomes of migrant construction workers, one (20.0%) to worsen the health outcomes, and one (20.0%) to not affect them. Mixed effects were found for employers failing to provide workers with protective equipment [26,27] and safety training [26,27,28] and workers’ compliance with safety rules [32,33]. Millward (2017) found that the passing of responsibilities between institutions that organized and delivered the 2022 FIFA World Cup in Qatar over the associated migrant workers’ occupational deaths and injuries was likely to lead to additional health damages suffered by migrant workers in Qatar. On the contrary, Zerguine and colleagues (2018) found no statistically significant association between migrant workers’ perception over the employing company’s interest in the adequacy of the overall work equipment and occupational injuries suffered by migrant workers.

## 4. Discussion

The selected studies included 41,745 participants, out of which at least 23,307 were construction workers. Overall, 14 studies (15.6%) investigated the effects of health hazards on the health outcomes of construction workers, out of which five (45.5% of studies eligible for search 1) were retrieved through search 1, while 10 (12.7% of studies eligible for search 2) were retrieved through search 2, considering that one study [17] was eligible to both searches. Five studies assessed the effect of lack of safety training on occupational injuries suffered by workers, with two studies (40.0%) finding an adverse effect and three (60.0%) a null effect. Three studies assessed the effect of employers not providing protective equipment on occupational injuries and fatalities of workers, with two studies (66.6%) finding an adverse effect and one (33.3%) a null effect. Three studies assessed the effect of employers pressing workers to rush through work on occupational injuries and fatalities of workers, with two studies finding a null effect (66.6%) and one (33.3%) an adverse effect. Three studies assessed the effect of workers’ perceptions of safety practices and employers’ positive commitments to safety on occupational injuries of workers, with two studies (66.6%) finding an inverse effect and one (33.3%) a null effect. Three studies assessed the effect of workers’ compliance with safety rules on occupational injuries and fatalities of workers, with two studies (66.6%) finding a beneficial effect and one (33.3%) a null effect. Two studies assessed the effect of not providing clear work instructions on occupational injuries and fatalities of workers, with one study (50%) finding a null effect and one (50%) an adverse effect. Two studies assessed the effect of workers fearing losing the job and worrying over their own financial situation on occupational injuries and fatalities of workers, with both studies (100%) finding a null effect. The effect of the following factors on occupational injuries and fatalities of workers was assessed by one study each (100%): an adverse effect was found for workers’ paying a labor recruiter, occurrence of mega-sporting events, passing of responsibilities between key actors over occupational injuries and fatalities, and environmental heat; a null effect was found for abusive language used by managers, unhygienic food, high skills required to conduct the job, unionization status, sleep deprivation, and overall work equipment not provided to workers; a beneficial effect was found for workers participating in voluntary safety activities, and an inverse effect was found for workers’ perceptions of worksite pollution and proper managerial leadership.

This is the first review systematically investigating the health outcomes of construction workers involved in building infrastructure for mega-sporting events. We used a narrative synthesis methodology to ensure transparent reporting of the synthesis of effect estimates. Meta-analyses were not conducted. We conducted two searches of the literature and included both peer-reviewed and non-peer-reviewed studies to comprehensively identify the hazards linked to the health outcomes of construction workers and migrant construction workers building infrastructure for mega-sporting events. Search 2 focused specifically on migrant construction workers as this population is designated as being both highly represented in the construction for mega-sporting events as well as more vulnerable to health risks [6].

### 4.1. Implications for Future Studies

The results from this review indicate that the majority of evidence on the health outcomes of construction workers involved in building infrastructure for mega-sporting events stems from studies using a cross-sectional design (nine studies, 64.3%), with only one study using a longitudinal design (7.2%). Although these cross-sectional studies provide valuable insights, they come with inherent limitations in establishing clear cause–effect relationships. Additionally, when considering the specific subset of migrant workers, these study designs may not effectively control for the potential influences of pre-migration health conditions on post-migration health outcomes. Therefore, caution should be exercised in making conclusive interpretations based solely on these studies. Further research is warranted to deepen our understanding of the key factors contributing to poorer health outcomes among migrant construction workers involved in mega projects [6,7]. However, understanding the effects of these events on the health of migrant workers can be challenging. The intermittent and short-term nature of mega-sporting events, the short-term visas and occupational status held by workers (many of whom may rapidly return to their place of origin upon conclusion of the project), and the exclusion of migrant workers from national health systems and governmental registries, all contribute to make it hard for researchers to study workers’ health outcomes and the effects of policy measures systematically. We suggest that these difficulties may at least be partially overcome by ethically applying new methods of data collection such as using electronic data and smartphones, and by urging contractors and governments to strengthen their reporting. In turn, this could allow much needed longitudinal and comparative research to be conducted within the context of mega-sporting events. Longitudinal studies are necessary to account for the potential influences of pre-migration health conditions on post-migration health outcomes. Comparative studies are necessary as they can provide a comprehensive understanding of the influence of cross-cultural differences and contextual factors on the health outcomes of migrant construction workers. When it comes to migrant construction workers, we suggest that future research should focus on all phases of migration—premigration, movement, arrival, integration, and return—in order to better understand the factors that affect migrant workers’ health and the obligations of governments and private companies that manage their stay. Governments, policy makers and contractors are responsible for improving the health outcomes of migrant construction workers under their commitments to the United Nations sustainable development goals to ‘leave no-one behind’ and ensuring healthcare coverage for all. They should support relevant future research endeavors by calling for, commissioning, and funding research aimed at improving policies on occupational health and safety for migrant construction workers. With the temporary nature of migrant workers taking part in the preparation of such events, the broad range of stakeholders must be better held to account.

The International Labour Organization’s conventions on occupational health, such as C155 and C167, and on migrant workers, such as the Migration for Employment Convention (C97) and the Migrant Workers (Supplementary Provisions) Convention (C143), could provide a valuable framework for addressing the health risks faced by workers in these projects. We recommend future research to investigate how adherence to these standards impacts worker health outcomes and how enforcement can be improved. Furthermore, longitudinal and comparative studies could assess the effectiveness of incorporating ILO standards into national policies governing mega-sporting events. For example, exploring the relationship between compliance with ILO guidelines and reduced occupational injuries among construction workers could provide actionable insights for policy development.

### 4.2. Limitations

The studies included in this review demonstrate a large variation in outcome measures and intervention types and we thus refrained from conducting meta-analyses. Given this heterogeneity, we opted for a narrative synthesis by following the Synthesis Without Meta-Analysis (SWiM) reporting guidelines, the state-of-the-art procedure for the transparent reporting of systematic reviews when quantitative means of data synthesis are not applicable. However, while this method enabled a comprehensive review of the findings, it may have limited the ability to draw measurement-based conclusions. The variability in study designs, outcome measures, interventions means, and lack of suitable evidence for meta-analyses led to synthesizing the evidence in a narrative format, which may not provide the same level of accuracy as a meta-analysis would. As such, the conclusions drawn should be considered with caution, particularly regarding the generalizability of the evidence across the diverse range of studies included.

### 4.3. Integration of Recent Evidence Post-Search

Subsequent to our searches, we identified new studies that provide insights into the health outcomes of migrant workers involved in mega-sporting events. Alzoubi et al. (2024) [35] highlighted how time constraints and financial pressures in large-scale projects, combined with inadequate oversight, create fertile ground for exploitation and worsened health outcomes in migrant construction workers. The study identified many factors enabling health-threatening practices including the kafala system, passport confiscation, debt bondage, contract substitution, salary abuse, and weak internal control mechanisms. These findings were supported by Richardson (2022) and Fachrul (2024) [36,37], who echoed similar concerns. These findings underscore the significant health risks faced by migrant construction workers, particularly in the context of mega-sporting events.

Hamidi (2022) [38] found that migrant construction workers were often employed illegally to build infrastructure for the 2024 Paris Olympics, enduring substandard living conditions and lacking the rights and protections afforded to legal employees. Similarly, Human Rights Watch (2022) [39] reported that migrant construction workers for the 2022 Beijing Winter Olympics faced arbitrary detention, forced labor, and torture. Additional and forthcoming studies on mega-sporting events and migrant construction workers should be systematically assessed in forthcoming research.

## 5. Conclusions

This review includes 11 studies on the health outcomes of workers involved in construction for mega-sporting events and 79 studies on violations, recruitment, and/or workplace practices relevant to migrant construction workers, commonly employed in construction for mega-sporting events. Overall, 20 health hazards were identified. These findings underscore the critical need to address the health and safety of migrant construction workers, particularly in the context of mega-sporting events.

Given the unique challenges faced by this vulnerable group, including short-term employment, exclusion from national health systems, and varying regulatory standards, it is essential to adopt a comprehensive, multi-phase approach to understanding and mitigating their risks. We recommend that forthcoming studies prioritize longitudinal and comparative studies that examine all phases of the migrant worker journey, including pre-migration, movement, arrival, integration, and return. We recommend that these studies focus on the specific health risks at each stage and evaluate the effectiveness of current interventions.

For policy makers, international sports organizations, and contractors, the findings highlight the urgent need for coordinated efforts to create robust health and safety standards that protect migrant workers in mega-sporting event construction. By implementing inclusive policies, improving labor recruitment practices, and ensuring the consistent enforcement of worker protections, stakeholders can enhance the well-being of this workforce. These efforts should be informed by evidence-based research, which will allow for the development of effective interventions, the establishment of best practices, and more informed decision-making that prioritizes worker health and well-being.

## Figures and Tables

**Table 1:** Inclusion and exclusion criteria for the eligibility screening of the retrieved articles based on the Population, Intervention, Comparison, Outcome, and Study (PICOS) framework.

PICOS Item	Search 1		Search 2	
	Inclusion Criterion	Exclusion Criterion	Inclusion Criterion	Exclusion Criterion
Participants	People	No people among participants	Migrant construction workers	No migrant construction worker among participants
Interventions	Involved in construction for mega-sporting events	No participant is involved in construction for mega-sporting events	Involved in construction, not necessarily for mega-sporting events	No participant is involved in construction
Comparisons	N/A	N/A	N/A	N/A
Outcomes	Health outcomes	No health outcome is addressed	Violations, recruitment and/or workplace practices are addressed	No violation, recruitment or workplace practice is addressed
Study characteristics	Any *	None	Any *	None

Acronyms, in order of appearance: N/A for not applicable. Notes: * Literature reviews were deemed an eligible article type considering that exploratory literature screenings conducted by the authors indicated that approximately half of the published literature in the scope of search 1 consisted of literature reviews.

**Table 2:** Features of the eligible studies stratified by systematic search.

		Overall n = 89	Search 1 n = 11	Search 2 n = 78 *
Country of focus	USA	21 (23.6%)	0 (0.0%)	21 (26.9%)
	Qatar	12 (13.5%)	2 (18.2%)	10 (12.8%)
	China	10 (11.2%)	1 (9.0%)	9 (11.5%)
	India	8 (9.0%)	0 (0.0%)	8 (10.3%)
	United Kingdom	6 (6.7%)	3 (27.3%)	3 (3.9%)
	Other countries	29 (32.6%)	2 (18.2%)	27 (34.6%)
	No particular country	3 (3.4%)	3 (27.3%)	0 (0.0%)
Type of study	Cross-sectional	68 (76.4%)	5 (45.4%)	63 (80.8%)
	Literature review	12 (13.5%)	4 (36.4%)	8 (10.2%)
	Longitudinal	5 (5.6%)	0 (0.0%)	5 (6.4%)
	Mixed	2 (2.2%)	0 (0.0%)	2 (2.6%)
	Retrospective	2 (2.2%)	2 (18.2%)	0 (0.0%)
Source of declared funding	Governments	37 (41.6%)	0 (0.0%)	37 (47.4%)
	No funding reported	35 (39.3%)	6 (54.5%)	29 (37.2%)
	Public donations	7 (7.9%)	0 (0.0%)	7 (9.0%)
	Foundations	5 (5.6%)	2 (18.2%)	3 (3.9%)
	Many sources	4 (4.5%)	2 (18.2%)	2 (2.5%)
	Inter-governmental organizations	1 (1.1%)	1 (9.1%)	0 (0.0%)
Risk of bias	Unclear	59 (66.3%)	3 (27.3%)	56 (71.8%)
	Low	17 (19.1%)	3 (27.3%)	14 (18.0%)
	High	7 (7.9%)	2 (18.1%)	5 (6.4%)
	Moderate	6 (6.7%)	3 (27.3%)	3 (3.8%)
Publication type	Peer-reviewed	65 (73.0%)	7 (63.6%)	58 (74.4%)
	Non peer-reviewed	24 (27.0%)	4 (36.4%)	20 (25.6%)

* Millward (2017) [17] was found to be eligible for both searches; therefore, it was counted only once under search 1 and omitted from search 2 statistics.

**Table 3:** Descriptive information of eligible studies addressing health outcomes in persons involved in the construction for mega-sporting events.

First Author’s Surname and Publication Year	Study Design	Sample Size	Sample Characteristics	Year, Type and Location of Mega-Sporting Events	Health Hazards Addressed	Health Outcomes Observed or Just Addressed	Direction of Effect	Risk of Bias
Bell 2015 [20]	Literature review	N/A	N/A	None specifically	None specifically	Proper project management skills are suggested to play a key role in protecting and improving the well-being and the health of construction workers. However, it is unclear whether any of the publications found by the review study address the well-being of workers involved in the construction for mega-sporting events.	N/A	Moderate
Bottecchia 2013 [18]	Cross-sectional	1200	Construction workers	2014 FIFA World Cup, Brasil	HBsAg	8 male participants (0.7% of the sample) were found HBsAg positive, had a mean age of 50 years, and none of them had heard about viral hepatitis before the study.	N/A	Low
Flouris 2019 [21]	Cross-sectional	125	91 migrant construction workers, 34 migrant agricultural workers, all males	2022 FIFA World Cup, Qatar	Sleep deprivation and exceptionally high environmental heat	On average, participants operating in the business-as-usual scenario worked for 60% of their time at normal core temperature levels (i.e., 36.5–37.4 °C), 30% at borderline-hyperthermic levels (37.5–37.9 °C), and 5% at hyperthermic levels (38.0–38.4 °C). At the same time, the same participants spent 40% of their time in breaks and 60% working mostly at a low intensity. Many of the participants working in the night suffered of insufficient sleep.	Of environmental heat: negative	Unclear
Flouris 2021 [6]	Retrospective	N/A	Total labor force at the national or regional level	Summer Olympic Games of 1992 in Spain, 1996 in the US, 2000 in Australia, 2004 in Greece, 2012 in the UK, 2016 in Brasil	None specifically	The incidence of occupational fatalities increased in the five years before each of the games was opened.	Of the occurring of mega-sporting events: negative	Moderate
Katsakiori 2008 [19]	Cross-sectional	63	labor inspectors	2004 summer Olympic Games, Greece	This study aimed at identifying health hazards associated with occupational fatalities	Occupational fatalities are likely to be caused by many factors acting in concert and the causing factors are primarily external to the worker and include the missed provision of appropriate protective equipment and clear information on the job assignment to the worker and overly tight work schedules, among others.	Of no protective equipment available: negative Of failing to use the available protective equipment: negative Of ambiguities from management and difficulty of the task: negative Of the time pressure from management: negative Of being new to a situation in the workplace: negative	Low
Millward 2017 [17]	Literature review	N/A	N/A	2022 FIFA World Cup, Qatar	Mismanagement of construction workers by the Government of Qatar, FIFA, World Cup sponsors, building contractors and sub-contractors, and recruitment agencies	The Government of Qatar, FIFA, World Cup sponsors, building contractors and sub-contractors and recruitment agencies are responsible for the adverse occupational health outcomes suffered by migrant construction workers, however each of the above actors passes on responsibility by framing the situation as regrettable but unconnected to them and thus the situations many migrant workers face continue unabated.	Of key actors passing responsibilities between them: negative	Moderate
Onarheim 2021 [7]	Literature review	N/A	N/A	None specifically	None specifically	No health outcomes observed, however recommendations to policy makers, international sports bodies and the construction industry are produced for promoting the health and access to healthcare of migrant construction workers in the upcoming mega-sporting events.	N/A	Low
Shanmugaratnam 2012 [22]	Cross-sectional	614	329 migrant construction workers, 285 native construction workers, 91% male	2012 summer Olympic and Paralympic Games, the UK	STIs	20 participants (3.3% of the sample) were infected with Chlamydia trachomatis and one (0.2%) with HBsAg while the remaining majority (96.5%) was STI-free.	N/A	Unclear
Shiplee 2011 [24]	Retrospective	N/A	N/A	2012 summer Olympic and Paralympic Games, the UK	None specifically	Lower accident rates among construction workers laboring at the Olympic and Paralympic Games site compared to the average of the British construction employment thanks to a described set of managerial activities spanning from the design of the construction program to its implementation.	Of proper program design, implementation and managerial leadership: positive	High
Sun 2008 [23]	Cross-sectional	40	Construction experts	2008 summer Olympic Games, China	This study aimed at identifying health hazards inherent in the construction sites of the 2008 Beijing Olympic venues.	Most of the identified health hazards were related to contractors and sub-contracts failing their safety responsibilities towards workers such as by missing to issue an emergency response plan and allowing safety rules to be broken under schedule pressure. However, the construction sites subjected to evaluation were found to be relatively safe overall (i.e., for a risk score ranging between 0 meaning no risk of adverse occupational health outcome to 100 meaning extremely high risk, the risk scores for the two sites were 16.1 and 18.3, respectively).	N/A	Unclear
Waterman 2007 [25]	Literature review	N/A	N/A	2012 summer Olympic and Paralympic Games, the UK	None specifically	Practices aimed at improving health outcomes in workers involved in the construction for mega-sporting events are described and they include, for instance, conducting hazard assessments and producing risk reduction plans prior to the implementation of a construction project.	N/A	High

Acronyms, in order of appearance: N/A stands for not available or not applicable, HBsAg for hepatitis B virus, STI for sexually transmitted disease.

## Data Availability

The data supporting the findings of this systematic review are derived from publicly available sources cited in the manuscript.

## Supplementary Materials

The following supporting information can be downloaded at: https://www.mdpi.com/article/10.3390/ijerph22010004/s1, Table S1: The study’s Preferred Reporting Items for Systematic Reviews and Meta-Analyses (PRISMA) checklist; Search algorithms used for search 1; Search algorithms used for search 2; Table S2: Synthesis Without Meta-analysis (SWiM) items checklist; List of studies included in the systematic review for search 1; List of studies included in the systematic review for search 2; Table S3: Descriptive information of studies investigating violations, recruitment, and/or workplace practices relevant to migrant construction workers.
