# Supplementary material for: Health Outcomes of Construction Workers Building Infrastructure for Mega-Sporting Events: A Systematic Review of the Literature
Source: Int J Environ Res Public Health. 2024 Dec 24;22(1):4. doi: 10.3390/ijerph22010004 (PMC11765195; doi:10.3390/ijerph22010004)
Supplement: Supplementary file 1 [file ijerph-22-00004-s001.zip › ijerph-3315867-supplementary.pdf]

SUPPLEMENTARY FILE

Health outcomes of construction workers building infrastructure for mega-sporting events: a systematic review of the literature

Davide J Testa,[1] João P Vale,[2,3] Leonidas G Ioannou,[1] Petros C Dinas,[1] Tiago S Mayor,[2,3] Kristine H Onarheim,[4] Zahra R Babar,[5] Sally Hargreaves,[6] Andreas D Flouris[1]

[1] Department of Physical Education and Sport Science, University of Thessaly, Karies, 42100 Trikala, Greece, Davide J Testa PhD Candidate, Leonidas G Ioannou Postdoctoral Researcher, Petros C Dinas Postdoctoral Researcher, Andreas Flouris Associate Professor

[2] CEFT, Transport Phenomena Research Centre, Faculty of Engineering, University of Porto, Dr. Roberto Frias, 4200-465 Porto, Portugal, João P Vale PhD Candidate, Tiago S Mayor Associate Professor

[3] ALiCE, Associate Laboratory in Chemical Engineering, Faculty of Engineering, University of Porto, Dr. Roberto Frias, 4200-465 Porto, Portugal, João P Vale PhD Candidate, Tiago S Mayor Associate Professor

[4] Department of Global Public Health and Primary Care, University of Bergen, Bergen Norway, Kristine H Onarheim Affiliated Researcher

[5] Center for International and Regional Studies, Georgetown University, Doha, Qatar, Zahra R Babar Director for Research

[6] Migrant Health Research Group, Institute for Infection and Immunity, University of London, Cranmer Terrace, SW17 London, UK, Sally Hargreaves Associate Professor

Corresponding author:

Andreas D. Flouris

FAME Laboratory

Department of Physical Education and Sport Science

University of Thessaly

Karies, Trikala, 42100, Greece

e-mail: andreasflouris@gmail.com

ORCID ID: 0000-0002-9823-3915

Table of contents

Table S1 4

Search algorithms used for search 1 8

Search algorithms used for search 2 8

Table S2.....

Table S3 10

List of studies included in the systematic review for search 1 13

List of studies included in the systematic review for search 2 14

Table S1. The study's Preferred Reporting Items for Systematic Reviews and Meta-Analyses (PRISMA) checklist.

Section and Topic	Item #	Checklist item	Appearing at/in page/table/figure
**TITLE**			
Title	1	Identify the report as a systematic review.	1
**ABSTRACT**			
Abstract	2	See the PRISMA 2020 for Abstracts checklist.	3, 4
**INTRODUCTION**			
Rationale	3	Describe the rationale for the review in the context of existing knowledge.	5, 6
Objectives	4	Provide an explicit statement of the objective(s) or question(s) the review addresses.	6, 7
**METHODS**			
Eligibility criteria	5	Specify the inclusion and exclusion criteria for the review and how studies were grouped for the syntheses.	8, 9, 10, Supplementary material
Information sources	6	Specify all databases, registers, websites, organisations, reference lists and other sources searched or consulted to identify studies. Specify the date when each source was last searched or consulted.	7, 8
Search strategy	7	Present the full search strategies for all databases, registers and websites, including any filters and limits used.	7, 8, Supplementary Material
Selection process	8	Specify the methods used to decide whether a study met the inclusion criteria of the review, including how many reviewers screened each record and each report retrieved, whether they worked independently, and if applicable, details of automation tools used in the process.	8, Supplementary Material
Data collection process	9	Specify the methods used to collect data from reports, including how many reviewers collected data from each report, whether they worked independently, any processes for obtaining or confirming data from study investigators, and if applicable, details of automation tools used in the process.	9
Data items	10a	List and define all outcomes for which data were sought. Specify whether all results that were compatible with each outcome domain in each study were sought (e.g. for all measures, time points, analyses), and if not, the methods used to decide which results to collect.	9, 10

Table S1. The study's Preferred Reporting Items for Systematic Reviews and Meta-Analyses (PRISMA) checklist.

Section and Topic	Item #	Checklist item	Appearing at/in page/table/figure
	10b	List and define all other variables for which data were sought (e.g. participant and intervention characteristics, funding sources). Describe any assumptions made about any missing or unclear information.	9, 10
Study risk of bias assessment	11	Specify the methods used to assess risk of bias in the included studies, including details of the tool(s) used, how many reviewers assessed each study and whether they worked independently, and if applicable, details of automation tools used in the process.	8
Effect measures	12	Specify for each outcome the effect measure(s) (e.g. risk ratio, mean difference) used in the synthesis or presentation of results.	9
Synthesis methods	13a	Describe the processes used to decide which studies were eligible for each synthesis (e.g. tabulating the study intervention characteristics and comparing against the planned groups for each synthesis (item #5)).	9
	13b	Describe any methods required to prepare the data for presentation or synthesis, such as handling of missing summary statistics, or data conversions.	9, 10
	13c	Describe any methods used to tabulate or visually display results of individual studies and syntheses.	9, 10
	13d	Describe any methods used to synthesize results and provide a rationale for the choice(s). If meta-analysis was performed, describe the model(s), method(s) to identify the presence and extent of statistical heterogeneity, and software package(s) used.	9, 10
	13e	Describe any methods used to explore possible causes of heterogeneity among study results (e.g. subgroup analysis, meta-regression).	9
	13f	Describe any sensitivity analyses conducted to assess robustness of the synthesized results.	Not available
Reporting bias assessment	14	Describe any methods used to assess risk of bias due to missing results in a synthesis (arising from reporting biases).	Not available
Certainty assessment	15	Describe any methods used to assess certainty (or confidence) in the body of evidence for an outcome.	Not available
**RESULTS**			
Study selection	16a	Describe the results of the search and selection process, from the number of records identified in the search to the number of studies included in the review, ideally using a flow diagram.	10, Figure 1, Figure 2

Table S1. The study's Preferred Reporting Items for Systematic Reviews and Meta-Analyses (PRISMA) checklist.

Section and Topic	Item #	Checklist item	Appearing at/in page/table/figure
	16b	Cite studies that might appear to meet the inclusion criteria, but which were excluded, and explain why they were excluded.	Not available
Study characteristics	17	Cite each included study and present its characteristics.	Table 2, Table 3
Risk of bias in studies	18	Present assessments of risk of bias for each included study.	Table 2, Table 3
Results of individual studies	19	For all outcomes, present, for each study: (a) summary statistics for each group (where appropriate) and (b) an effect estimate and its precision (e.g. confidence/credible interval), ideally using structured tables or plots.	Table 2, Table 3, Figure 3
Results of syntheses	20a	For each synthesis, briefly summarise the characteristics and risk of bias among contributing studies.	12, 14, 15, 16, Figure 3
	20b	Present results of all statistical syntheses conducted. If meta-analysis was done, present for each the summary estimate and its precision (e.g. confidence/credible interval) and measures of statistical heterogeneity. If comparing groups, describe the direction of the effect.	Not applicable
	20c	Present results of all investigations of possible causes of heterogeneity among study results.	10, 11, Table 1
	20d	Present results of all sensitivity analyses conducted to assess the robustness of the synthesized results.	Not available
Reporting biases	21	Present assessments of risk of bias due to missing results (arising from reporting biases) for each synthesis assessed.	Not available
Certainty of evidence	22	Present assessments of certainty (or confidence) in the body of evidence for each outcome assessed.	Not available
**DISCUSSION**			
Discussion	23a	Provide a general interpretation of the results in the context of other evidence.	17, 18
	23b	Discuss any limitations of the evidence included in the review.	19, 20
	23c	Discuss any limitations of the review processes used.	20
	23d	Discuss implications of the results for practice, policy, and future research.	19, 20

Table S1. The study's Preferred Reporting Items for Systematic Reviews and Meta-Analyses (PRISMA) checklist.

Section and Topic	Item #	Checklist item	Appearing at/in page/table/figure
**OTHER INFORMATION**			
Registration and protocol	24a	Provide registration information for the review, including register name and registration number, or state that the review was not registered.	7
	24b	Indicate where the review protocol can be accessed, or state that a protocol was not prepared.	7
	24c	Describe and explain any amendments to information provided at registration or in the protocol.	Not available
Support	25	Describe sources of financial or non-financial support for the review, and the role of the funders or sponsors in the review.	22, 23
Competing interests	26	Declare any competing interests of review authors.	22
Availability of data, code and other materials	27	Report which of the following are publicly available and where they can be found: template data collection forms; data extracted from included studies; data used for all analyses; analytic code; any other materials used in the review.	7, 8, 9

Search algorithms used for search 1

In Embase, Ovid MEDLINE, PubMed:

(construction* OR building) AND (worker* OR laborer* OR labourer* OR mason* OR constructor*) AND (soccer OR tennis OR basketball OR stadium OR cup OR games OR sport* event* OR Olympic)

In Scopus:

TITLE-ABS-KEY ((construction* OR building) AND (worker* OR laborer* OR labourer* OR mason* OR constructor*) AND (soccer OR tennis OR basketball OR stadium OR cup OR games OR sport* event* OR Olympic))

Search algorithms used for search 2

In Google Scholar:

(construction OR building OR industry) AND (worker OR workers OR laborer OR laborers OR labourer OR labourers OR mason OR masons OR constructor OR constructors OR migrant OR migrants) AND (kafala OR violation OR violations OR "health card" OR "ethical recruitment")

In Embase, Ovid MEDLINE:

(construction* OR building OR construction industr*.tw) AND (worker* OR labo?rer* OR mason? OR constructor? OR migrant*) AND (kafala OR violation* OR health card* OR ethical recruitment)

In PubMed:

(construction* OR building OR “construction industr*.tw”) AND (worker* OR labo?rer* OR mason? OR constructor? OR migrant*) AND (kafala OR violation* OR “health card*” OR “ethical recruitment”)

In Scopus:

TITLE-ABS-KEY ((construction* OR building OR “construction industr*.tw”) AND (worker* OR labo?rer* OR mason? OR constructor? OR migrant*) AND (kafala OR violation* OR “health card*” OR “ethical recruitment”))

Table S2. Synthesis Without Meta-analysis (SWiM) items checklist.

SWiM reporting item	Item description	Page in manuscript where item is reported	Additional notes
1 Grouping studies for synthesis	1a Provide a description of, and rationale for, the groups used in the synthesis (e.g., groupings of populations, interventions, outcomes, study design)	9, 10	
	1b Detail and provide rationale for any changes made subsequent to the protocol in the groups used in the synthesis	Not available	No changes in the grouping made subsequent to the protocol publication
2 Describe the standardised metric and transformation methods used	Describe the standardised metric for each outcome. Explain why the metric(s) was chosen and describe any methods used to transform the intervention effects, as reported in the study, to the standardised metric, citing any methodological guidance consulted	9	
3 Describe the synthesis methods	Describe and justify the methods used to synthesise the effects for each outcome when it was not possible to undertake a meta-analysis of effect estimates	9	
4 Criteria used to prioritise results for summary and synthesis	Where applicable, provide the criteria used, with supporting justification, to select the particular studies, or a particular study, for the main synthesis or to draw conclusions from the synthesis (e.g., based on study design, risk of bias assessments, directness in relation to the review question)	9	

5 Investigation of heterogeneity in reported effects	State the method(s) used to examine heterogeneity in reported effects when it was not possible to undertake a meta-analysis of effect estimates and its extensions to investigate heterogeneity	9	
6 Certainty of evidence	Describe the methods used to assess the certainty of the synthesis findings	Not available	In line with previous systematic reviews where authors undertook vote counting for data synthesis, a Grading of Recommendations, Assessment, Development and Evaluations (GRADE) assessment was not conducted
7 Data presentation methods	7a Describe the graphical and tabular methods used to present the effects (e.g., tables, forest plots, harvest plots)	9, 10	
	7b Specify key study characteristics (e.g., study design, risk of bias) used to order the studies, in the text and any tables or graphs, clearly referencing the studies included	9, 10	
8 Reporting results	For each comparison and outcome, provide a description of the synthesised findings and the certainty of the findings. Describe the result in language that is consistent with the question the synthesis addresses, and indicate which studies contribute to the synthesis	10, 11, 12, 13, 14, 15,16, 17	

9 Limitations of the synthesis	Report the limitations of the synthesis methods used and/or the groupings used in the synthesis and how these affect the conclusions that can be drawn in relation to the original review question	20	

List of studies included in the systematic review for search 1, in alphabetical order by first author's surname:

1. Bell N, Powell C, Sykes P. Securing the well-being and engagement of construction workers: An initial appraisal of the evidence. In: Raidén AB, Aboagye-Nimo E, editors. Annual ARCOM Conference 20152015. p. 489-98.
2. Bottecchia M, Do Ó K, Moraes M: Screening for hepatitis B virus in Maracanã workers. Journal of Viral Hepatitis. 2013; 20:16-42.
3. Flouris AD, Ioannou L, Dinas P, Mantzios K, Gkiata P, Gkikas G, et al.: Assessment of occupational heat strain and mitigation strategies in Qatar. 2019.
4. Flouris AD, Babar Z, Ioannou LG, Onarheim KH, Phua KH, Hargreaves S: Improving the evidence on health inequities in migrant construction workers preparing for big sporting events. 2021; 374:n1615. 10.1136/bmj.n1615 %J BMJ.
5. Katsakiori P, Manatakis E, Goutsos S, Athanassiou G: Factors attributed to fatal occupational accidents in a period of 5 years preceding the Athens 2004 Olympic Games. International journal of occupational safety and ergonomics: JOSE. 2008; 14; 3:285-92. 10.1080/10803548.2008.11076766.
6. Millward P: World Cup 2022 and Qatar's construction projects: Relational power in networks and relational responsibilities to migrant workers. Current Sociology. 2017; 65; 5:756-76. 10.1177/0011392116645382.
7. Onarheim KH, Phua KH, Babar ZR, Flouris AD, Hargreaves S: Health and social needs of migrant construction workers for big sporting events. 2021; 374:n1591. 10.1136/bmj.n1591 %J BMJ.
8. Shanmugaratnam S, Horne P, Coyne KM: Olympic outreach: testing for sexually transmitted infections in construction workers. International journal of STD & AIDS. 2012; 23; 9:659-60. 10.1258/ijsa.2012.012033.

9. Shiplee H, Waterman L, Furniss K, Seal R, Jones J, editors. Delivering London 2012: health and safety. Proceedings of the Institution of Civil Engineers-Civil Engineering; 2011: Thomas Telford Ltd.
10. Sun Y, Fang D, Wang S, Dai M, Lv X: Safety Risk Identification and Assessment for Beijing Olympic Venues Construction. 2008; 24; 1:40-7. doi:10.1061/(ASCE)0742-597X(2008)24:1(40).
11. Waterman L: London 2012: occupational health in the construction programme. The journal of the Royal Society for the Promotion of Health. 2007; 127; 3:113-8. 10.1177/1466424007077343.

List of studies included in the systematic review for search 2, in alphabetical order by first author's surname:

1. Abdul-Aziz A-R. 2001. Foreign workers and labour segmentation in malaysia's construction industry. Construction Management and Economics 19:789-798.
2. Al-Kaabi N, Hadipriono F. 2003. Construction safety performance in the united arab emirates. Civil Engineering and Environmental Systems 20:197-212.
3. Amnesty International. 2009. Disposable labour: Rights of migrant workers in south korea. London, United Kingdom:Amnesty International.
4. Amnesty International. 2010. Trapped: The exploitation of migrant workers in malaysia. London, United Kingdom:Amnesty International.
5. Amnesty International. 2011. False promises: Exploitation and forced labour of nepalese migrant workers. London, United Kingdom:Amnesty International.
6. Amnesty International. 2013. The dark side of migration: Spotlight on qatar's construction sector ahead of the world cup. London, United Kingdom:Amnesty International.
7. Amnesty International. 2014. No extra time: How qatar is still failing on workers' rights ahead of the world cup. London, United Kingdom:Amnesty International.

8. Amnesty International. 2015. Promising little, delivering less: Qatar and migrant labour abuse ahead of the 2022 football world cup. London, United Kingdom:Amnesty International.
9. Amnesty International. 2016. The ugly side of the beautiful game: Exploitation of migrant workers on a qatar 2022 world cup site. London, United Kingdom:Amnesty International.
10. Anand V. 1998. Advocating for the rights of construction workers: Nirman's experience. Indian Journal of Social Work 59:847-863.
11. Anderson JT, Hunting KL, Welch LS. 2000. Injury and employment patterns among hispanic construction workers. Journal of occupational and environmental medicine 42:176-186.
12. Arcury TA, Mills T, Marín AJ, Summers P, Quandt SA, Rushing J, et al. 2012. Work safety climate and safety practices among immigrant latino residential construction workers. American journal of industrial medicine 55:736-745.
13. Bernhardt A, Spiller MW, Polson D. 2013a. All work and no pay: Violations of employment and labor laws in chicago, los angeles and new york city. Social Forces 91:725-746.
14. Bernhardt A, Spiller MW, Theodore N. 2013b. Employers gone rogue: Explaining industry variation in violations of workplace laws. 66:808-832.
15. Berntsen L. 2016. Reworking labour practices: On the agency of unorganized mobile migrant construction workers. 30:472-488.
16. Cedillo L, Lippel K, Nakache D. 2019. Factors influencing the health and safety of temporary foreign workers in skilled and low-skilled occupations in canada. New solutions : a journal of environmental and occupational health policy : NS 29:422-458.
17. Chan APC, Javed AA, Lyu S, Hon CKH, Wong FKW. 2016. Strategies for improving safety and health of ethnic minority construction workers. 142:05016007.

18. Chan APC, Javed AA, Wong FKW, Hon CKH, Lyu S. 2017. Evaluating the safety climate of ethnic minority construction workers in hong kong. 143:04017006.
19. Dainty A, Gibb AG, Bust P, Goodier C. 2007. Health, safety and welfare of migrant construction workers in the south east of england.1-54.
20. Dhal M. 2020. Labor stand: Face of precarious migrant construction workers in india. 146:04020048.
21. Diop A, Trung Le K, Ewers MC. 2016. Working and living conditions of migrant workers in the gcc. In: India migration report 2016: Gulf migration, (Irudaya Rajan S, ed), 1-318.
22. Dong X, Ringen K, Men Y, Fujimoto A. 2007. Medical costs and sources of payment for work-related injuries among hispanic construction workers. Journal of occupational and environmental medicine 49:1367-1375.
23. Dutta MJ. 2017. Migration and health in the construction industry: Culturally centering voices of bangladeshi workers in singapore. 14:132.
24. Evia C. 2011. Localizing and designing computer-based safety training solutions for hispanic construction workers. 137:452-459.
25. Evia C, Patriarca A. 2012. Beyond compliance:Participatory translation of safety communication for latino construction workers. 26:340-367.
26. Fernandes D, G.D BP. 2011. Social networks of migrant construction workers in goa. Indian Journal of Industrial Relations 47:65+.
27. Forst L, Ahonen E, Zanoni J, Holloway-Beth A, Oschner M, Kimmel L, et al. 2013. More than training: Community-based participatory research to reduce injuries among hispanic construction workers. American journal of industrial medicine 56:827-837.
28. Friberg JH. 2010. Working conditions for polish construction workers and domestic cleaners in oslo: Segmentation, inclusion and the role of policy. In: A continent moving west? Eu enlargement and labour migration from central and eastern europe, (Black R, Engbersen G, Okólski M, Panţîru C, eds):Amsterdam University Press.

29. Gardner AM. 2010. City of Strangers: Gulf Migration and the Indian Community in Bahrain. 1 ed:Cornell University Press.
30. Gardner A, Pessoa S, Diop A, Al-Ghanim K, Le Trung K, Harkness L. 2013. A portrait of low-income migrants in contemporary qatar. Journal of Arabian Studies 3:1-17.
31. Hallowell MR, Yugar-Arias IF. 2016. Exploring fundamental causes of safety challenges faced by hispanic construction workers in the us using photovoice. Safety Science 82:199-211.
32. Hamid W, Tutt D. 2019. "Thrown away like a banana leaf": Precarity of labour and precarity of place for tamil migrant construction workers in singapore. Construction Management and Economics 37:513-536.
33. Hare B, Cameron I, Real KJ, Maloney WF. 2013. Exploratory case study of pictorial aids for communicating health and safety for migrant construction workers. 139:818-825.
34. Hassan HA, Houdmont J. 2014. Health and safety implications of recruitment payments in migrant construction workers. Occupational medicine (Oxford, England) 64:331-336.
35. Human Rights Watch. 2006. United arab emirates: Building towers, cheating workers : Exploitation of migrant construction workers in the united arab amirates:Human rights watch (HRW).
36. Human Rights Watch. 2009. "Are you happy to cheat us?": Exploitation of migrant construction workers in russia:Human Rights Watch.
37. Human Rights Watch. 2012. Building a better world cup: Protecting migrant workers in qatar ahead of fifa 2022:Human Rights Watch.
38. Hussain R, Pedro A, Lee DY, Pham HC, Park CS. 2020. Impact of safety training and interventions on training-transfer: Targeting migrant construction workers. International journal of occupational safety and ergonomics : JOSE 26:272-284.

39. Jayaram N, Varma D. 2020. Examining the ‘labour’ in labour migration: Migrant workers’ informal work arrangements and access to labour rights in urban sectors. The Indian Journal of Labour Economics 63:999-1019.

40. Jiang Y, Luo H, Yang F. 2020. Influences of migrant construction workers’ environmental risk perception on their physical and mental health: Evidence from china. 17:7424.

41. Kim J-M, Son K, Yum S-G, Ahn S. 2020. Analyzing the risk of safety accidents: The relative risks of migrant workers in construction industry. 12:5430.

42. Ling FYY, Dulaimi MF, Chua M. 2013. Strategies for managing migrant construction workers from china, india, and the philippines. 139:19-26.

43. Loganathan S, Kalidindi SN. 2016. Absenteeism and turnover of migrant construction workers in indian projects—a survey-based study. In: Construction research congress 2016, 1793-1802.

44. Lyu S, Hon CKH, Chan APC, Wong FKW, Javed AA. 2018. Relationships among safety climate, safety behavior, and safety outcomes for ethnic minority construction workers. International journal of environmental research and public health 15.

45. Malit FT, Tsourapas G. 2021. Migration diplomacy in the gulf – non-state actors, cross-border mobility, and the united arab emirates. Journal of Ethnic and Migration Studies 47:2556-2577.

46. McGlothlin J, Hubbard B, Aghazadeh F, Hubbard S. 2009. Case study: Safety training issues for hispanic construction workers. Journal of occupational and environmental hygiene 6:D45-50.

47. Mendelsohn JB, Calzavara L, Light L, Burchell AN, Ren J, Kang L. 2015. Design and implementation of a sexual health intervention for migrant construction workers situated in shanghai, china. Emerging themes in epidemiology 12:16.

48. Menzel NN, Shrestha PP. 2012. Social marketing to plan a fall prevention program for latino construction workers. American journal of industrial medicine 55:729-735.

49. Millward P. 2017. World cup 2022 and qatar’s construction projects: Relational power in networks and relational responsibilities to migrant workers. Current Sociology 65:756-776.
50. Nagayama T. 1992. Clandestine migrant workers in japan. Asian and Pacific migration journal : APMJ 1:623-636.
51. Nissen B, Angee A, Weinstein M. 2008. Immigrant construction workers and health and safety:The south florida experience. 33:48-62.
52. Nungsari M, Flanders S, Chuah H-Y. 2020. Poverty and precarious employment: The case of rohingya refugee construction workers in peninsular malaysia. Humanities and Social Sciences Communications 7:120.
53. O'Connor T, Loomis D, Runyan C, Abboud dal Santo J, Schulman M. 2005. Adequacy of health and safety training among young latino construction workers. Journal of occupational and environmental medicine 47:272-277.
54. Patel A, Giri J. 2019. Climate change, migration and women: Analysing construction workers in odisha. 49:97-113.
55. Pattanaik BK. 2009. Young migrant construction workers in the unorganised urban sector. 29:19-40.
56. Peoples Union for Democratic Rights. 2009. In the name of national pride: Blatant violation of workers' rights at the commonwealth games construction site. Delhi, India.
57. Rathod JM. 2016. Danger and dignity: Immigrant day laborers and occupational risk. Seton Hall law review 46:813-882.
58. Regueiro R. 2020. Shared responsibility and human rights abuse: The 2022 world cup in qatar. Tilburg Law Review.
59. Robertson C, Kerr M, Garcia C, Halterman E. 2007. Noise and hearing protection: Latino construction workers' experiences. AAOHN journal : official journal of the American Association of Occupational Health Nurses 55:153-160.

60. Roelofs C, Sprague-Martinez L, Brunette M, Azaroff L. 2011. A qualitative investigation of hispanic construction worker perspectives on factors impacting worksite safety and risk. Environmental Health 10:84.
61. Ruttenberg R, Lazo M. 2004. Spanish-speaking construction workers discuss their safety needs and experiences.
62. Shantz J. 2011. Discrimination against latin american workers during pre-olympic games construction in vancouver. Employee Responsibilities and Rights Journal 23:75-80.
63. Sippola M, Kall K. 2016. Locked in inferiority? The positions of estonian construction workers in the finnish migrant labour regime. In: Labour mobility in the enlarged single european market, Vol. 32:Emerald Group Publishing Limited, 215-240.
64. Sönmez S, Apostolopoulos Y, Tran D, Rentrope S. 2011. Human rights and health disparities for migrant workers in the uae. Health and human rights 13:E17-35.
65. Swider S. 2015. Building china: Precarious employment among migrant construction workers. 29:41-59.
66. Theodore N, Valenzuela A, Meléndez E. 2009. Worker centers: Defending labor standards for migrant workers in the informal economy. International Journal of Manpower 30:422-436.
67. Theodoropoulou I. 2020. Blue-collar workplace communicative practices: A case study in construction sites in qatar. Language Policy 19:363-387.
68. Thörnqvist C, Bernhardsson S. 2015. Their own stories – how polish construction workers posted to sweden experience their job situation, or resistance versus life projects. 21:23-36.
69. Torres R, Heyman R, Munoz S, Apgar L, Timm E, Tzintzun C, et al. 2013. Building austin, building justice: Immigrant construction workers, precarious labor regimes and social citizenship. Geoforum 45:145-155.

70. Tutt D, Dainty A, Gibb A, Pink S. 2011. Migrant construction workers and health & safety communication. Norfolk, United Kingdom:Construction Industry Training Board - Construction Skills.
71. United Nations General Assembly. 2020. Visit to qatar: Report of the special rapporteur on contemporary forms of racism, racial discrimination, xenophobia and related intolerance.
72. Vinck P, Pham PN, Fletcher LE, Stover E. 2009. Inequalities and prospects: Ethnicity and legal status in the construction labor force after hurricane katrina. 22:470-478.
73. Voivozeanu A. 2019. Precarious posted migration: The case of romanian construction and meat-industry workers in germany. Central and Eastern European Migration Review 8:1-<p>99</p>.
74. Wang T, Li Y, Li G. 2016. Case study of integrated prefab accommodations system for migrant on-site construction workers in china. Journal of Professional Issues in Engineering Education and Practice 142:05016005.
75. Wu C, Luo X, Wang T, Wang Y, Sapkota B. 2020. Safety challenges and improvement strategies of ethnic minority construction workers: A case study in hong kong. International Journal of Occupational Safety and Ergonomics 26:80-90.
76. Yang D. 2020. Why don’t they complain? The social determinants of chinese migrant workers’ grievance behaviors. 73:366-392.
77. Yeoh BSA, Baey G, Platt M, Wee K. 2017. Bangladeshi construction workers and the politics of (im)mobility in singapore. City 21:641-649.
78. Zerguine H, Tamrin SBM, Jalaludin J. 2018. Prevalence, source and severity of work-related injuries among "foreign" construction workers in a large malaysian organisation: A cross-sectional study. Industrial health 56:264-273.
79. Zhang SX, Spiller MW, Finch BK, Qin Y. 2014. Estimating labor trafficking among unauthorized migrant workers in san diego. The Annals of the American Academy of Political and Social Science 653:65-86.

Table S3. Descriptive information of studies investigating violations, recruitment, and/or workplace practices relevant to migrant construction workers.

First author's surname[a] and year of publication	Study design	Sample characteristics	Sample size	Recruitment practices addressed	Violations addressed	Workplace practices addressed	Effects on health outcomes observed	Direction of effect	Risk of bias
Abdul-Aziz 2001	Cross-sectional	Employers, foreign and local workers at construction sites in Malaysia	2,168	N/A	Workers' violations of occupational safety rules<br>Employers failing to provide proper work equipment and instructions to workers	Wage inequalities between local and migrant workers<br>On-site accommodation for workers lacking basic services such as access to electricity<br>Extra-work services provided by subcontracting employers to migrant workers lurking the latter into financial dependence to the former	N/A	N/A	Unclear

Al-Kaabi 2003	Cross-sectional	Construction contractors in the UAE	121	N/A	Providence by contractors of individual and collective safety equipment to workers<br>Providence and maintenance by contractors of basic hygiene at the construction site<br>Providence by contractors of trained personnel for safety monitoring at the construction site<br>Contractors keeping a record of all occupational injuries occurred	Insurances provided by contractors to workers<br>Training provided by contractors to workers prior the start of the job	N/A	N/A	Unclea r

Amnesty International 2009	Cross-sectional	Migrant workers in South Korea, workers' union representatives, factory owners and managers, staff of foreign embassies in South Korea, migrant centers and other NGOs, the National Human Rights Commission of Korea and the South Korean government	> 60 migrant workers, unreported size of the other sub-samples	Recruitment of migrant workers via brokers leading to the indebtedness and exploitation of migrant workers<br>Recruitment of migrant workers via governments leading to less debts but longer time spent by migrant workers waiting to receive a VISA<br>The South Korean government imposing negative HIV testing as a requirement for migrant workers to be hired in South Korea	Recruiters failing to inform prospective migrant workers of the details of their employment abroad such as their remuneration<br>Arrest, detention and deportation of migrant workers by the police against law enforcement procedures<br>Migrant workers' wage withholding<br>Employers failing to provide health and safety equipment to migrant workers and training in a language understandable by migrant workers	Inequalities in the rights enjoyed by local versus migrant workers<br>Lack of governmental monitoring of workplaces for safety and contractual violations at the damage of migrant workers<br>Irregular migrant workers being not eligible to be covered by the national health insurance while regular migrant workers and locals are	Migrant workers perceived that the lack of health and safety training in a language that they were proficient in was the main cause of the greater prevalence of occupational injuries suffered by migrants compared to locals<br>Suggested that migrant workers suffered a greater prevalence of occupational injuries compared to locals because the formers were employed where the working conditions were poorer, the safety provision inadequate and	Lack of health and safety training : null	Unclear

					Migrant workers being not allowed to join or form trade unions		locals refused to work		
Amnesty International 2010	Cross-sectional	Migrant workers in Malaysia including construction workers, employers, recruitment agents, lawyers, staff of governmental organisations and NGOs, diplomatic missions and religious groups	> 200 migrant workers, unreported size of the other sub-samples	Recruitment process of migrant workers	Violations at the damage of migrant workers regarding their recruitment, physical integrity, wage, work hours, health and safety training, travel and work documentation, free will when it comes to labouring and the occurring of occupational injuries and deaths	N/A	N/A	N/A	Unclear

Amnesty International 2011	Cross-sectional and retrospective	Nepali migrant workers in Nepal having laboured abroad or planning to do so, brokers, staff of foreign employment agencies, recruitment agencies, local NGOs, workers' union representatives, United Nations agencies, the National Human Rights Commission and the Nepali government	N/A	Recruitment process of migrant workers	Violations at the damage of migrant workers regarding their recruitment, wage, work hours, travel documentation and free will when it comes to labouring	N/A	N/A	N/A	Unclea r

Amnesty International 2013	Cross-sectional	Migrant workers in Qatar including construction workers, employers, independent experts, journalists, academics, representatives of migrant communities in Qatar, staff of foreign embassies in Qatar, FIFA, the National Human Rights Committee of Qatar, the Qatar Foundation for Combating Human Trafficking, the Hamad Medical Corporation and the Qatari government	289 migrant workers, unreported size of the other sub-samples	N/A	Violations at the damage of migrant workers regarding their recruitment, wage, work hours, travel and work documentation, free will when it comes to labouring and the occurring of occupational injuries and deaths	N/A	N/A	N/A	Unclear

Amnesty International 2014	Literature review	N/A	N/A	Progress made by the Qatari government in addressing issues with the recruitment of migrant construction workers in Qatar	Progress made by the Qatari government in addressing wage withholding and forced labour at the damage of migrant construction workers	Progress made by the Qatari government in improving migrant construction workers' labour conditions and their access to justice and healthcare, and allowing them to join trade unions and leave Qatar at their will	N/A	N/A	Modera te
Amnesty International 2015	Literature review	N/A	N/A	Progress made by the Qatari government in addressing issues with the recruitment of migrant construction workers in Qatar	Progress made by the Qatari government in addressing wage withholding and passport confiscation at the damage of migrant construction workers	Progress made by the Qatari government in improving migrant construction workers' labour conditions and their access to justice and healthcare, and allowing them to join trade unions and leave Qatar at their will	N/A	N/A	Low

Amnesty International 2016	Cross-sectional	Migrant workers in Qatar, staff of contracting and sub-contracting companies and the Qatari government	234 migrant workers including construction workers, unreported size of the other sub-samples	N/A	Violations at the damage of migrant workers regarding their recruitment, wage, travel documentation and free will when it comes to labouring<br>Responsibilities of employers, FIFA and the Qatari government on the observed violations at the damage of migrant workers	N/A	N/A	N/A	Unclear

Anand 1998	Literature review	N/A	N/A	Channels through which migrant construction workers are recruited	Migrant construction workers getting less than the minimum wage<br>Migrant constructions workers not being provided with health and safety equipment<br>Women construction workers being paid less compared to men<br>Women construction workers being denied of governmental support in case of maternity	Migrant construction workers getting paid with seasonal frequency<br>Women construction workers being more likely to labour as unskilled workers compared to men<br>Women construction workers usually delivering babies at the construction site	Migrant construction workers suffered chronic health conditions due to the exposure to dangers at the worksite and the lack of health and safety equipment, training and work-related instructions	Lack of health and safety equipment: null<br>Lack of health and safety training: null<br>Lack of health and safety instructions: null	High

Anderson 2000	Longitudinal	Construction workers of African, Hispanic and white ethnicity having suffered occupational injuries in the US	N/A	N/A	N/A	Employment patterns of migrant construction workers across ethnicities	Suggested that ethnicity is a predictor of trade of employment within the construction sector with Hispanics workers being over-represented among the general labourers, and trade and workers' union status are predictors of non-fatal occupational injuries with not-unionized general labourers being more likely to suffer such injuries	Level of work skills require d to conduc t the job: null<br>Unioniz ation status: null	Unclea r

Arcury 2012	Cross-sectional and longitudinal	Hispanic construction workers in the US	119	N/A	N/A	Occupational safety measures put in place by employers as perceived by migrant construction workers across construction trades and how they associated with workers' perceived work safety climate<br><br>Occupational safety behaviours put in place by migrant construction workers and how they are associated with workers' perceived work safety climate	N/A	N/A	Unclea r

Bernhardt 2013a	Cross-sectional	Low-wage workers in the US including construction workers	4,387	N/A	Violation of low-wage workers' rights<br>Association between low-wage workers' features including migration background and workplace violations	N/A	N/A	N/A	Unclea r
Bernhardt 2013b	Cross-sectional	Low-wage workers in the US including construction workers	4,387	N/A	Violation of low-wage workers' rights across industries including construction	N/A	N/A	N/A	Unclea r
Berntsen 2016	Cross-sectional	Local and migrant construction workers in the Netherlands	71	Recruitment process of migrant construction workers	Violations of migrant construction workers' rights and actions put in place by migrants to redress them	Lower unionization among migrant construction workers compared to locals or migrant workers in other industries	N/A	N/A	Unclea r

Cedillo 2019	Cross-sectional	Labourers, NGOs spoke-persons and workers' union representatives all operating in Canada	99	N/A	Wage, hour and training violations committed by employers at the damage of migrant construction workers	N/A	N/A	N/A	Unclea r
Chan 2016	Cross-sectional	Occupational safety professionals and experts with experience in managing ethnic minority workers	40	N/A	N/A	Strategies that could be implemented by employers and work-site managers in order to improve the health and safety of ethnic minority construction workers	N/A	N/A	Unclea r

Chan 2017	Cross-sectional	Nepali and Pakistani construction workers in China	320	N/A	N/A	Occupational safety behaviours put in place by migrant construction workers and how they are associated with workers' perceived work safety climate	The statistical model used to test whether migrant construction workers' health and safety perceptions and behaviours predicted injury occurrence at work in the same workers was found to be not statistically significant	Health and safety percept ions: null<br>Health and safety behavi ours: null	Unclea r

Dainty 2007	Cross-sectional	Migrant construction workers, employers and other stakeholders in the UK	84	Recruitment process of migrant construction workers	N/A	Work roles covered by migrant construction workers<br>Racism, cultural discrimination and wider communication practices at the worksite among migrant construction workers<br>Health and safety awareness, attitudes and training practices among migrant construction workers	N/A	N/A	Unclea r

Dhal 2020	Cross-sectional	Internal migrant construction workers in India	84	N/A	Employers committing wage violations at the damage of migrant construction workers	Wage amount of migrant construction workers<br>Absence of wage inequalities between female and male migrant construction workers<br>Migrant construction workers working without having eaten for days due to a lack of financial resources partially resulting from rare and short-termed employment opportunities<br>Abusive language used by contractors towards migrant	N/A	N/A	Unclea r

						construction workers<br><br>Marginal support delivered by trade unions and the Indian government to migrant construction workers			

Diop 2016	Cross-sectional	Migrant workers in Bahrain, Kuwait, Oman, Qatar and Saudi Arabia	3,600	N/A	N/A	Wage payment practices by employers<br>Benefits on top of a wage given by employers to migrant workers including sick leave days and health insurance coverage<br>Migrant workers daily hours of work per day<br>Workplace practices that workers perceive as challenges or are satisfied with	N/A	N/A	Unclea r

Dong 2007	Cross-sectional	Construction workers in the US including Hispanic workers	7,025	N/A	N/A	Hispanic construction workers being less likely to be unionized compared to their white, non-Hispanic counterparts<br>Hispanic construction workers paying more out of their pocket on average for covering medical expenses arising from work-related injuries compared to their white, non-Hispanic counterparts	N/A	N/A	Low

Dutta 2017	Cross-sectional	Bangladeshi construction workers in Singapore	60	N/A	N/A	Migrant construction workers purposefully avoiding to think about health and safety risks at work in order to not be scared and continue working and providing an income to their families<br>Migrant construction workers continuing to work despite debilitating occupational injuries in order to continue providing money to their families in the absence of paid sick leave<br>Employers' productivity demands towards migrant	Migrant construction workers perceived that the productivity demands from employers led them to rush with their work and to suffer occupational injuries as a consequence<br>Migrant construction workers reported that they had skipped meals because the food they had received was stale or unhygienic in turn leading to lethargy and consequently to occupational injuries<br>Migrant construction workers reported that	Time pressure from management: null<br>Unhygienic food: null<br>Sleep deprivation: null<br>Abusive language from management: null<br>Worries over financial situation: null	Unclear

						construction workers leading the latter to rush with their work<br><br>Migrant construction workers receiving poor-quality or unhygienic food from catering companies leading workers to skip meals<br><br>Migrant construction workers labouring lethargically due to insufficient sleep<br><br>Supervisors using abusive language towards migrant construction workers<br><br>Faulty actions at work by migrant construction	they laboured lethargically due to the insufficient sleep at night leading in turn to occupational injuries<br><br>Migrant construction workers reported that supervisors were using abusive language and this often resulted in the workers being upset at work and to suffer occupational injuries<br><br>Migrant construction workers reported worrying while working about managing to pay debts back and keep sending remittances to		

						workers due to worries on paying debts back and managing to send remittances to their families	their families and this in turn leading to occupational injuries		
Evia 2011	Cross-sectional	Hispanic construction workers in the US	N/A	N/A	N/A	Health and safety training practices tailored to the needs of Hispanic construction workers	N/A	N/A	Unclea r
Evia 2012	Cross-sectional	Hispanic construction workers in the US	> 31	N/A	N/A	Practices for the development of health and safety communication products tailored to the needs of Hispanic construction workers	N/A	N/A	High

Fernandes 2011	Cross-sectional	Migrant construction workers, contractors and sub-contractors all operating in India	> 53 migrant construction workers, unreported size of the other sub-samples	Identification of the profile of people through which migrant construction workers manage to get recruited	N/A	Health and safety training practices put in place by migrant construction workers and their supervisors<br>Flow of credit between migrant construction workers and contractors	N/A	N/A	Unclear
Forst 2013	Longitudinal	Migrant construction workers in the US including Hispanic workers	463	N/A	N/A	Health and safety training practices tested on Hispanic construction workers	N/A	N/A	Unclear

Friberg 2010	Cross-sectional	Polish migrant workers in Norway including construction workers	510	Practices for the recruitment of Polish construction workers	Minimum wage violations committed by employers at the damage of Polish construction workers	Perceived access to the Norwegian state's welfare for sick pay among unlawfully-employed Polish construction workers compared to their lawfully-employed counterparts<br>Wage and hour inequalities between Polish and local construction workers	N/A	N/A	Unclea r
Gardner 2010	Cross-sectional	Migrant workers in Bahrain including construction workers	66	Recruitment process of migrant workers and their subjugation under the Kafala system	N/A	N/A	N/A	N/A	Unclea r

Gardner 2013	Cross-sectional	Low-wage migrant workers in Qatar including construction workers	1,189	Recruitment process of migrant workers under the Kafala system	Violations committed by employers at the damage of migrant workers including passport confiscation and wage withholding, among others	Wage amount of migrant workers across national, ethnical and religious groups	N/A	N/A	Unclea r

Hallowell 2016	Cross-sectional	Hispanic construction workers in the US	17	N/A	N/A	Hispanic construction workers perceiving themselves as being more focused at working fast rather than safe and systematically assigned more dangerous activities compared to their local counterparts, and unwilling to accept negative feedback at work<br><br>Hispanic construction workers finding difficult to follow work instructions because available in English or in a poor Spanish	N/A	N/A	Low

Hamid 2019	Cross-sectional	Indian construction workers in Singapore	11	N/A	N/A	Wage inequalities between Indian construction workers and other groups of construction workers	N/A	N/A	Unclea r
Hare 2013	Cross-sectional	Migrant construction workers in the UK	50	N/A	N/A	Health and safety communication practices tested on migrant construction workers	N/A	N/A	Low

Hassan 2014	Cross-sectional	Migrant construction workers in an unreported country in the Middle East	651	Payment to labour recruiters by migrant construction workers in order to land a job and how the payment is associated to the likelihood for migrant construction workers to experience occupational injuries	N/A	N/A	Skilled migrant construction workers that had paid labour recruiters were more likely to have had experienced occupational injuries in the previous 12 months while no statistically significant associations were found for unskilled workers and supervisors	Paying a labour recruiter: negative	Low
Human Rights Watch 2006	Cross-sectional	60 migrant construction workers in the UAE, employers, UAE government officials, lawyers, journalists, health professionals and foreign diplomats	60 migrant workers, unreported size of the other sub-samples	Recruitment process of migrant construction workers	Violations at the damage of migrant construction workers regarding their recruitment, wage, travel documentation and the occurring of occupational injuries and deaths	N/A	N/A	N/A	Unclear

Human Rights Watch 2009	Cross-sectional	Migrant construction workers in Russia, officials of the Kyrgyz, Russian and Tajik governments, diplomats of other governments, employers, recruiters, staff of employment agencies, intermediary employment agencies and NGOs	146 migrant workers, unreported size of the other sub-samples	N/A	Violations at the damage of migrant construction workers regarding their recruitment, physical integrity, wage, travel and work documentation, free will when it comes to labouring and the occurring of occupational injuries and deaths	N/A	N/A	N/A	Unclear

Human Rights Watch 2012	Cross-sectional	Migrant construction workers and other-low wage workers in Qatar, academics, employers, camp supervisors, journalists, legal counselors, recruiting agents, staff of local community organizations, charity groups, the Qatar National Human Rights Committee, the Qatari government and foreign diplomatic institutions	114 between migrant construction workers [73], other low-wage workers, academics, employers, camp supervisors, journalists, legal counselors and recruiting agents, unreported size of	N/A	Violations at the damage of migrant construction workers regarding their recruitment, wage, housing, health and safety training, travel documentation, free will when it comes to labouring and the occurring of occupational injuries and deaths	N/A	N/A	N/A	Unclear

			the other sub-sampl es						
Hussain 2020	Cross-sectional	Migrant construction workers in Qatar	30	N/A	N/A	Health and safety training practices tested on migrant construction workers	N/A	N/A	Low

Jayaram 2020	Cross-sectional	Migrant workers in India including construction workers	≥ 694 includi ng 163 constr uction worker s	N/A	Wage withholding committed by employers at the damage of migrant construction workers during the pandemic<br>Migrant construction workers lacking medical treatment and sick pay after suffering occupational injuries<br>Employers keeping workers off the books and relating to migrant construction workers through many levels of intermediaries in order to sustainably abuse of migrant	Employment conditions of female and male migrant construction workers<br>Wage inequalities between male and female migrant construction workers<br>Lack of maternity benefits for migrant construction workers	N/A	N/A	Unclea r

					construction workers' rights				
Jiang 2020	Cross-sectional	Migrant construction workers in China	678	N/A	N/A	Self-precautionary health behaviours of migrant construction workers and how they are associated with their perception of worksite air, industrial waste and noise pollution	Migrant construction workers that had a greater perception of worksite pollution were statistically significantly mentally and physically healthier compared to their counterparts with a lower perception suggesting that a greater perception of worksite pollution is positively associated with self-precautionary behaviours in migrant construction workers	Perceiving air, industrial waste and noise pollution at the worksite: positive	Low

Kim 2020	Cross-sectional	Local and migrant construction workers having suffered occupational injuries in South Korea	1,766	N/A	N/A	Health and safety measures to be put in place by worksite managers in order to protect migrant construction workers' health	N/A	N/A	Unclea r
Ling 2013	Cross-sectional	Managers with experience in managing migrant construction workers in Singapore	≥ 32	N/A	N/A	Practices to effectively manage cultural diversity among migrant construction workers	N/A	N/A	Unclea r
Loganathan 2016	Cross-sectional	Managers and labour sub-contractors in India	36	N/A	N/A	Causes of absenteeism and turnover of migrant construction workers	N/A	N/A	Unclea r

Lyu 2018	Cross-sectional	Nepali and Pakistani construction workers in China	289	N/A	Ethnic minority workers’ safety compliance and how it is associated with worksite accidents	Ethnic minority workers’ safety climate and how it is associated with worksite accidents	Ethnic minority workers’ health and safety participation and compliance were found to be negatively associated with the same workers experiencing occupational injuries and near-misses	Health and safety particip ation: positive<br>Health and safety compli ance: positive	Unclea r
Malit 2021	Literature review	N/A	N/A	Role played by non-state actors in the definition of the recruitment process of migrant workers in the UAE	N/A	N/A	N/A	N/A	Modera te
McGlothlin 2009	Cross-sectional	Hispanic construction workers in the US	42	N/A	N/A	Prevalence of Hispanic construction workers having undertook formal health and safety training	N/A	N/A	Unclea r

Mendelsohn 2015	Longitudinal	Migrant construction workers in China	848	N/A	N/A	Sexual-health education programs tested on migrant construction workers at their workplace	N/A	N/A	Low
Menzel 2012	Longitudinal	Local and Hispanic construction workers in the US	773	N/A	N/A	Social marketing practices aimed at improving Hispanic construction workers’ fall protection behaviours	N/A	N/A	Unclea r

Millward 2017	Literature review	N/A	N/A	N/A	Passing of responsibilities between the Qatari government, FIFA, World Cup sponsors, building contractors and sub-contractors and recruitment agencies over violations at the damage of migrant construction workers	N/A	The passing of responsibilities between responsible actors may cause migrant construction workers to keep suffering the same violations over time leading to occupational injuries and deaths	Key actors passin g respon sibilitie s betwee n them: negativ e	Modera te
Nagayama 1992	Literature review	N/A	N/A	Recruitment of migrant workers through illegal labour mediators requiring migrants to pay recruitment fees to be hired	Illegal arrangements made by employers to hire and pay migrant workers	Lack of protections granted by the Japanese government and trade unions to clandestine migrant workers	N/A	N/A	High

Nissen 2008	Cross-sectional	Local and migrant workers in the US	400	N/A	N/A	Associations between workers' unionization and documentation status and safety training received, use of personal protective equipment and other workplace practices	N/A	N/A	Low

Nungsari 2020	Cross-sectional	Rohingya refugee construction workers in Malaysia	314	N/A	N/A	Rohingya workers suffering late and lack of salary payments, ambiguous work schedule and periods of employment, lack of safety standards at workplace, physical and verbal abuse by employers, harassment by law enforcement and dirty and unhygienic quarters provided onsite by employers<br>Wage inequalities between local and Rohingya workers	N/A	N/A	Low

O'Connor 2005	Cross-sectional	Hispanic construction workers in the US	50	N/A	Hispanic workers under the age of 18 performing work tasks prohibited by law for people their age	Inadequate health and safety training provided by employers to Hispanic workers<br>Association between Hispanic workers' fluency in the English language and likelihood to receive health and safety training<br>Health and safety communication practices between employers and Hispanic workers	N/A	N/A	Unclea r

Patel 2019	Cross-sectional	Migrant construction workers in India	N/A	N/A	Maternity benefits not provided by employers to female migrant workers	Work conditions and career prospects of female migrant workers compared to the male counterpart	N/A	N/A	Unclea r
Pattanaik 2009	Cross-sectional	Migrant construction workers in India	1,200	Lack of a registration system of recruited migrant workers	Migrant workers not having access to government-subsidised food	Wage of migrant workers<br>Migrant workers not being provided with first-aid facilities and compensation against occupational accidents	N/A	N/A	Unclea r

PUDR 2009	Cross-sectional	Migrant construction workers in India	N/A	Recruitment process of migrant construction workers	Violations at the damage of migrant construction workers regarding their wage, accommodation, access to welfare and the availability and conditions of work equipment, and the role played by the Indian government in allowing such violations to happen	N/A	Suggested that the critical conditions of a crane used at the construction site and the failing of managers to have it fixed or replaced led to the death of a migrant construction worker	N/A	Unclear

Rathod 2016	Cross-sectional	Local and migrant workers in the US including construction workers	84	Assistance in the recruitment of migrant workers and protection of their rights by worker centres	Employers failing to provide personal protective equipment to migrant workers	Association between workers' unionization status affiliation to worker centres, and work speed and occupational health outcomes<br>Employer's threat to denounce migrant workers to the authorities following an occupational injury suffered by the latter in order to not pay the missing wage to workers	N/A	N/A	Low

Regueiro 2020	Literature review	N/A	N/A	N/A	Use of a shared responsibility framework involving the Qatari and Swiss governments and FIFA to grant protection and reparation to the migrant workers injured or killed in the construction of infrastructure for the 2022 FIFA World Cup	N/A	N/A	N/A	High
Robertson 2007	Cross-sectional	Hispanic construction workers in the US	15	N/A	N/A	Hispanic workers' perceptions over occupational noise and reasons to use or not hearing protections at work	N/A	N/A	Unclea r

Roelofs 2011	Cross-sectional	Hispanic construction workers in the US	18	N/A	Instances where employers failed at providing Hispanic workers with adequate work and personal protective equipment<br>Supervisors' firing Hispanic workers refusing to conduct work for which they had not been trained or while injured	Work supervisors pressing Hispanic workers to work faster than local workers<br>Illiteracy among Hispanic workers and consequent inability to read safety signals at construction sites	Hispanic workers perceived that greater occupational falls and death rates suffered by Hispanic workers compared to local workers at construction sites were positively associated with pressure from work supervisors to work fast and fear of losing the job if refusing to conduct work tasks unsafely, while no association was perceived between the greater accident rates and lack of health and safety training	Time pressure from management: null<br>Fear of losing job: null<br>Lack of health and safety training: null	Unclear

Ruttenberg 2004	Cross-sectional	Hispanic construction workers in the US	47	N/A	N/A	Providence of safety training and equipment to Hispanic workers<br>Effect of safety training on Hispanic workers' occupational safety behaviours<br>Hispanic workers' negative feedback on received safety training	N/A	N/A	Unclea r
Shantz 2011	Literature review	N/A	N/A	N/A	Hispanic workers' mistreatment by employers compared to Canadian and European workers on wage, accommodation , expenses and meal arrangements	N/A	N/A	N/A	High

Sippola 2016	Longitudinal	Estonian construction workers in Finland, staff of the Estonian and Finnish governments, foreign embassies and workers’ unions	29	Practices for the recruitment of Estonian workers in Finland	N/A	Wage inequalities between local and Estonian workers	N/A	N/A	Unclea r
Sönmez 2011	Literature review	N/A	N/A	Functioning of the Kafala sponsorship system for the recruitment of migrant workers in the UAE	N/A	Kafala system leading migrant workers to experience forced labour	N/A	N/A	Modera te
Swider 2015	Cross-sectional	Migrant construction workers in China, labour contractors, construction managers and officials of the Chinese government	83	Practices for the recruitment of internal migrant construction workers in China	N/A	N/A	N/A	N/A	Unclea r

Theodore 2009	Cross-sectional	Worker center executive directors and senior staff in the US	N/A	Recruitment of construction workers for day jobs through worker centers	N/A	Assistance provided by worker centers for redressing workers' grievances	N/A	N/A	Unclea r
Theodoropoulo u 2020	Cross-sectional	Migrant construction workers in Qatar	10	N/A	N/A	Communication practices between workers	N/A	N/A	Unclea r
Thörnqvist 2015	Cross-sectional	Polish construction workers in Sweden, workers' unions representatives and officials of a local government in Sweden	12	Recruitment of Polish workers to Sweden	Employers failing to provide Polish workers with wages according to law	Wage inequalities between local and Polish workers	N/A	N/A	Unclea r
Torres 2013	Cross-sectional	Construction workers in the US including migrants, and employers	332	N/A	Employers committing wage theft at the damage of migrant workers	Inadequate provision of information on workers' rights to migrant workers	N/A	N/A	Unclea r

Tutt 2011	Cross-sectional	Migrant construction workers and managers in the UK	N/A	N/A	N/A	Observed and recommended health and safety communication practices between migrant workers, employers, local co-workers	N/A	N/A	Unclea r

United Nations 2020	Cross-sectional	Staff of the Qatari government, representatives of the National Human Rights Committee and United Nations, academics and migrant workers including construction workers	N/A	N/A	Discriminative practices in the recruitment and police profiling of migrant workers along racial and nationality lines<br>Lack of access to healthcare by migrant workers	Employers blackmailing migrant workers of detention and deportation to get them to work in exploitative conditions<br>Migrant workers being disincentivized by law to report work violations suffered at the hand of employers and thus refraining to do so<br>Wage inequalities between migrant workers along nationality lines	N/A	N/A	Unclea r

Vinck 2009	Cross-sectional	Local and migrant construction workers in the US	212	N/A	Lack of access to healthcare by Hispanic workers	Wage inequalities between documented and undocumented workers, and between African-American, Hispanic and white workers	N/A	N/A	Low
Voivozeanu 2019	Cross-sectional	Romanian workers in Germany including construction workers	32	Recruitment patterns of Romanian workers to Germany through posted work	Theft of migrant workers' wage at the hand of employers	N/A	N/A	N/A	Unclea r
Wang 2016	Cross-sectional	Migrant construction workers in China	N/A	N/A	N/A	Improved on-site accommodation for migrant workers	N/A	N/A	Unclea r

Wu 2018	Cross-sectional	Indian, Nepali and local construction workers in China, and representatives of workers' unions	69	N/A	Employers failing to provide migrants with working safety equipment	Communication patterns between migrant and local workers<br>Discrimination of migrant workers in work assignments and training opportunities along ancestry and nationality lines<br>Migrant workers' attitudes when comes to prioritizing earning over safety and working under the effect of drugs	N/A	N/A	Unclea r

Yang 2019	Cross-sectional	Migrant construction workers in China	4,000	N/A	N/A	Factors associated with migrant workers' grievance behaviours in response to workplace violations	N/A	N/A	Low
Yeoh 2017	Cross-sectional	Bangladeshi construction workers in Singapore	205	Recruitment patterns of Bangladeshi workers in Singapore	N/A	N/A	N/A	N/A	Unclea r

Zerguine 2018	Cross-sectional	Migrant construction workers in Malaysia	323	N/A	Migrant workers' access to safety training and equipment	Migrant workers' perception over employers' commitment on safety and health	Workers perceiving a higher employers' safety commitment and interest in overall safety and health and safety training were statistically significantly less likely to suffer occupational injuries and experience near-misses<br>No statistical association between workers' perceived adequacy of the overall work equipment and occupational injuries	Company's commitment into the health and safety of workers: positive<br>Company's interest into:<br>Health and safety of workers: positive<br>Health and safety equipment: null<br>Health and safety	Low

								training : positive<br>Overall work equipm ent: null	
Zhang 2014	Cross-sectional	Migrant workers in the US including construction workers	826	N/A	Human trafficking and workplace violations suffered by migrant workers including construction workers	N/A	N/A	N/A	Unclea r

[a]The name of the publishing institution rather than the first author's surname appears in the first column of the table when the list of authors is not provided in the relevant full-text article.

Acronyms, in order of appearance: UAE stands for the United Arab Emirates; NGO for non-governmental organisation; N/A for not available or not applicable; FIFA for Federation Internationale de Football Association; US for United States of America; UK for United Kingdom of Great Britain and Northern Ireland; PUDR for Peoples Union for Democratic Rights.
